# Noninvasive genomic detection of melanoma

**DOI:** 10.1111/j.1365-2133.2011.10239.x

**Published:** 2011-04

**Authors:** W Wachsman, V Morhenn, T Palmer, L Walls, T Hata, J Zalla, R Scheinberg, H Sofen, S Mraz, K Gross, H Rabinovitz, D Polsky, S Chang

**Affiliations:** * Research Service, VA San Diego Healthcare SystemSan Diego, CA 92161, U.S.A.; † Division of Hematology–Oncology and Moores Cancer Center, Department of Medicine, University of California San DiegoLa Jolla, CA, U.S.A.; ‡ Division of Dermatology, Department of Medicine, University of California San DiegoLa Jolla, CA, U.S.A.; § Therapeutics Clinical ResearchSan Diego, CA, U.S.A.; ¶ DermTech International, Inc.La Jolla, CA, U.S.A.; ** Dermatology AssociatesFlorence, KY, U.S.A.; †† Dermatologist Medical Group of North CountyOceanside, CA, U.S.A.; ‡‡ Dermatology Research AssociatesLos Angeles, CA, U.S.A.; §§ Solano Dermatology AssociatesVallejo, CA, U.S.A.; ¶¶ Skin Surgery Medical GroupSan Diego, CA, U.S.A.; *** Skin and Cancer AssociatesPlantation, FL, U.S.A.; ††† Departments of Dermatology and Pathology, New York University School of MedicineNew York, NY, U.S.A.

## Abstract

**Background:**

Early detection and treatment of melanoma is important for optimal clinical outcome, leading to biopsy of pigmented lesions deemed suspicious for the disease. The vast majority of such lesions are benign. Thus, a more objective and accurate means for detection of melanoma is needed to identify lesions for excision.

**Objectives:**

To provide proof-of-principle that epidermal genetic information retrieval (EGIR™; DermTech International, La Jolla, CA, U.S.A.), a method that noninvasively samples cells from stratum corneum by means of adhesive tape stripping, can be used to discern melanomas from naevi.

**Methods:**

Skin overlying pigmented lesions clinically suspicious for melanoma was harvested using EGIR. RNA isolated from the tapes was amplified and gene expression profiled. All lesions were removed for histopathological evaluation.

**Results:**

Supervised analysis of the microarray data identified 312 genes differentially expressed between melanomas, naevi and normal skin specimens (*P*<0·001, false discovery rate *q*<0·05). Surprisingly, many of these genes are known to have a role in melanocyte development and physiology, melanoma, cancer, and cell growth control. Subsequent class prediction modelling of a training dataset, consisting of 37 melanomas and 37 naevi, discovered a 17-gene classifier that discriminates these skin lesions. Upon testing with an independent dataset, this classifier discerned *in situ* and invasive melanomas from naevi with 100% sensitivity and 88% specificity, with an area under the curve for the receiver operating characteristic of 0·955.

**Conclusions:**

These results demonstrate that EGIR-harvested specimens can be used to detect melanoma accurately by means of a 17-gene genomic biomarker.

The occurrence of malignant melanoma has been rising for decades, leading to a doubling of the incidence rate over the last 10–20 years.^1^,^2^ The invasive form of the disease is already the seventh most common serious cancer in the U.S.A. with a lifetime risk of one in 41 in men and one in 61 in women. Once melanoma has disseminated, the patient's prognosis is dismal.^3^–^5^ Most deaths from melanoma could be prevented, however, if the disease were detected and excised at an early stage, while still confined to the skin.^4^ For example, *in situ* melanoma, the earliest skin stage, has a nearly 100% cure rate when adequately excised. Once melanoma has progressed locally, especially if it has invaded to a depth of 4 mm or more, the 10-year survival rate is < 50% because it has already metastasized before the skin lesion was excised. This makes the early detection of melanoma critical to patient survival.

Current clinical detection of melanoma relies upon visual cues, including the ‘ABCDE’ criteria for pigmented naevi, and results of optical imaging techniques like dermoscopy and confocal microscopy. Due to the diverse morphologies of pigmented lesions, early diagnosis of this tumour can be quite challenging.^4^,^6^ The occurrence of atypical naevi, precancerous lesions that can mimic the visual presentation of melanoma but do not have the histopathological features of this cancer, is a major confounder to the accurate clinical diagnosis of melanoma. Studies have shown that clinicians are only able to determine whether a suspicious pigmented lesion is melanoma or not with 54–90% sensitivity.^6^–^8^ Clinical expertise was found to be a key determinant of melanoma diagnostic accuracy.^8^ Using the benign-to-malignant ratio as an endpoint, researchers in Australia found that general practitioners biopsied approximately 30 benign pigmented lesions for every melanoma, while dermatologists biopsied more than 12 pigmented lesions for every melanoma they diagnosed.^9^ Taken together, health care professionals biopsy many pigmented lesions to detect a melanoma, and may also leave some melanomas undetected at their early stages.

Techniques to improve the clinical diagnosis of suspicious pigmented skin lesions are based on enhanced imaging methods such as dermoscopy and confocal microscopy. Dermoscopy has been shown to improve the sensitivity of melanoma detection by 10–27%;^10^,^11^ however, some early melanomas are missed by this technique.^12^ Other detection strategies include sequential digital epiluminescence microscopy, reflectance confocal microscopy, automated instrumentation for image analysis, MelaFind™ (Mela Sciences, Irvington, NY, U.S.A.), and the comparison of serial body photographs, e.g. of atypical naevi, taken at frequent intervals.^13^–^17^ Reflectance confocal microscopy, arguably the more accurate of the confocal techniques, does not reach a sensitivity of 100%.^18^ Although digital epiluminescence microscopy has been shown to improve the sensitivity of melanoma detection significantly, in particular for thin lesions, it has a specificity of < 20%.^14^

The current gold standard for diagnosing melanoma is histopathological examination of the excised tissue. This necessitates biopsy of the lesion, an invasive, time- and resources-consuming procedure that can be impractical, especially in those patients who have many dysplastic naevi. Moreover, even histopathology has its limitations. Due to its subjective nature, discordant readings between expert dermatopathologists are reported to occur in 10–35% of potential cases of melanoma.^19^,^20^

Epidermal genetic information retrieval (EGIR™; DermTech International, La Jolla, CA, U.S.A.) uses a custom adhesive film to sample RNA from stratum corneum noninvasively. RNA recovered from the surface of the skin by EGIR has been quantified using ribonuclease protection assay, quantitative real-time reverse transcription–polymerase chain reaction (qRT-PCR) and DNA microarray analysis to differentiate irritant from allergic skin reactions and by qRT-PCR to assess changes in gene expression in psoriatic skin lesions in response to biotherapy.^21^–^23^ These studies showed the feasibility of using EGIR-harvested RNA to assess differences in dermatopathology. In the current study, we sought to exploit this technology to determine whether the expression profile of RNA in stratum corneum of normal skin differs from that overlying a naevus or a malignant melanoma. We report here that analysis of such EGIR specimens has identified some 312 genes that differentiate normal skin and naevi from melanomas, many of which are relevant to the underlying pathology. Furthermore, we have used this EGIR-based strategy to develop a 17-gene classifier that detects both *in situ* and invasive melanoma with high accuracy.

## Materials and methods

### Patients and clinical protocols

The study protocols were reviewed and approved by the various Institutional Review Boards. Study subjects gave written informed consent prior to participation and the study was conducted according to the Declaration of Helsinki principles. All study subjects were 18 years or older and had a pigmented lesion 4 mm or greater in diameter that required biopsy due to suspicion for melanoma (Table 1). Study exclusion criteria included application of topical medications to the lesion or use of systemic steroids within 30 days of tape stripping; presence of a generalized skin disorder such as psoriasis, a photosensitivity disorder, or eczema; known allergy to tape or latex; use of sunscreen or topical moisturizer within 24 h of tape stripping; and lesions with clinically overt bleeding, ulceration or serous exudation. After informed consent, the suspicious pigmented lesion was taped stripped, as previously described,^23^ and then biopsied as per standard of care. If a lesion was smaller in size than the 17-mm diameter adhesive tape disc, the outer edge of the lesion was demarcated on the tape with indelible ink. A tape-stripped specimen was used in the study only after the histopathological diagnosis of the biopsied tissue was reviewed at the primary site and confirmed by the central dermatopathology reviewer. As a control, each subject's normal appearing skin was also sampled by tape stripping. Eighty-two per cent of these control specimens were garnered from the mastoid process, with the remainder coming from the upper back, and all were at a minimum distance of 15 cm from the suspicious lesion.

**Table 1:** Summary of patient information

	Melanoma (*n*=76)	Naevus (*n*=126)
Age (years), mean (range)	60·5 (26–95)	45·3 (19–79)
Female/male	27/49	58/68
Anatomical location		
Scalp	8	2
Face	13	3
Neck	3	2
Shoulder	14	26
Trunk	12	74
Upper limb	15	10
Lower limb and hip	11	9
Superficial spreading melanoma		
*In situ*	25	
Invasive	40	
Lentigo maligna melanoma		
*In situ*	5	
Invasive	5	
Nodular melanoma	1	
Benign naevi		51
Atypical naevi		75
Tumour stage		
Tis	31	
T1	35	
T2	8	
T3	1	
T4	1	

### Materials and reagents

The EGIR tape kit contains four small circular adhesive discs, each 17 mm in diameter, with a polyurethane backing. The tape was purchased from Adhesives Research (Glen Rock, PA, U.S.A.) and fabricated into discs with a polyurethane backing by Diagnostic Laminations Engineering (Oceanside, CA, U.S.A.). Universal human reference RNA was purchased from Stratagene (San Diego, CA, U.S.A.). Reverse transcriptase, Taqman Universal Master Mix, which included all buffers and enzymes necessary for the amplification and fluorescent detection of β-actin cDNA, was purchased from Applied Biosystems (Foster City, CA, U.S.A.). MELT total nucleic acid isolation system was purchased from Ambion (Austin, TX, U.S.A.). GeneChip human genome U133 plus 2.0 arrays were purchased from Affymetrix (Santa Clara, CA, U.S.A.).

### RNA isolation and quantification

All tape strips were processed in the laboratory at DermTech International. The RNA was extracted from tapes by means of MELT and quantified by TaqMan qRT-PCR for β-actin mRNA expression level, as per Wong *et al.*^22^ RNA quality was assessed by microfluidic electrophoretic analysis using an Experion Automated Electrophoresis Station (BioRad, Inc., Hercules, CA, U.S.A.).

### RNA amplification and array hybridization

RNA harvested from the EGIR tape strips was amplified using the Ovation Pico RNA Amplification System (NuGEN Technologies, Inc., San Carlos, CA, U.S.A.) and hybridized with Affymetrix human genome U133 plus 2.0 GeneChip, according to standard manufacturer protocols.

### Gene expression analysis

The image files from scanning the Affymetrix GeneChips with the Affymetrix series 3000 scanner were converted to CEL-format files using the Affymetrix GeneChip Operating Software version 1.4. Normalization of GeneChip CEL files was carried out using the GCRMA software from Bioconductor (http://www.bioconductor.org). After filtering out for background and low expressed genes (level < 100 for a gene target across all samples), data were imported into GeneSpring (Agilent, Santa Clara, CA, U.S.A.). A supervised analysis was performed to identify genes differentially expressed between pigmented lesions (both melanoma and naevi) (biopsy proven) and control, nonlesional skin specimens. This was performed by anova with multiple testing correction using the Westfall and Young permutation method^24^ [*P*<0·001, false discovery rate (FDR) *q*<0·05].

Cluster analysis was performed according to Eisen *et al.*^25^ Data were first log2 transformed and then median centred for genes and samples. The resulting normalized data were further analysed by the self-organizing map algorithm^26^ and then clustered with Spearman rank correlation similarity metrics.

To develop a melanoma detection classifier, only the GeneChip data from melanomas and naevi were used. These were randomly divided into a training set used for multigene classifier selection and a test set for evaluating the performance of the resultant classifier. Differentially expressed genes were identified by unpaired *t*-test and multiple testing correction^24^ (*P*<0·05, *q*<0·05) from a training set of 37 melanomas and 37 naevi. This group of genes was then mined by stochastic gradient boosting, as developed by Friedman^27^,^28^ (available for use as TreeNet®; Salford Systems, San Diego, CA, U.S.A.) to develop a class prediction model. The resultant multigene classifier was validated with an independent test dataset composed of 39 melanomas and 89 naevi.

Gene ontology and pathway analysis was performed with Ingenuity Pathway Analysis (IPA) system software version 8.5 (Ingenuity Systems, Inc., Redwood City, CA, U.S.A.). Genes with their corresponding identifiers and fold change values were uploaded for interrogation. After analysis, significance of the biological functions and the canonical pathways was tested by the Fisher's exact test *P*-value to determine the probability that each biological/canonical pathway assigned to the dataset is due to chance alone.

## Results

### Use of epidermal genetic information retrieval-harvested RNA to identify genes differentially expressed between melanoma, naevi and normal skin

**Fig 1:**
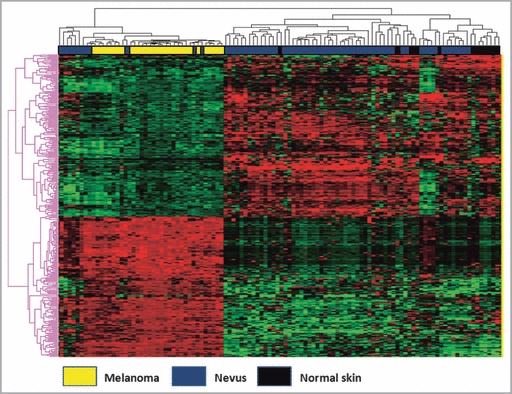
Hierarchical clustering analysis of differentially expressed genes among melanomas, naevi and normal skin. These 312 genes determined from microarray analysis of epidermal genetic information retrieval specimens differentiate melanoma from atypical naevi and normal skin (*P*<0·001, false discovery rate *q*<0·05).

In this study, we sought to address whether the EGIR method can be used to discern between melanoma, naevi and normal skin. We harvested 29 melanomas (*in situ* and invasive), 68 naevi (benign and atypical) and 15 normal skin specimens from which RNA was isolated, amplified and profiled on the Affymetrix U133 plus 2.0 GeneChip. anova analysis (*P*<0·001, Westfall and Young permutation multiple testing correction,^24^ FDR *q*<0·05) of the resulting microarray data identified 312 differentially expressed genes (the annotated list of genes is provided in Table S1; see Supporting Information). Hierarchical clustering analysis of these 312 genes showed two major branches in the heat map (Fig. 1). The branch on the left side of the heat map contained all 29 melanomas and 13 of the naevi and was clearly separated from the right branch that contained the bulk of the naevi as well as all of the normal skin specimens. Most of the 13 naevi that grouped together with melanomas were Clark (also known as ‘dysplastic’) naevi with mild to severe cytological atypia, and as such bear some morphological resemblance to melanoma. Of note is that normal skin specimens could not be separated from naevi based on the expression of these 312 genes, suggesting that the RNA in the stratum corneum over these lesions is similar. Most of the naevi that grouped together with normal skin were nondysplastic, compound, intradermal or junctional naevi. Taken together, these findings demonstrate that expression profiling of EGIR-harvested RNA from stratum corneum can be used to identify genes differentially expressed between melanoma, naevi and normal skin.

### Biological functions of the 312 genes related to melanoma

**Fig 2:**
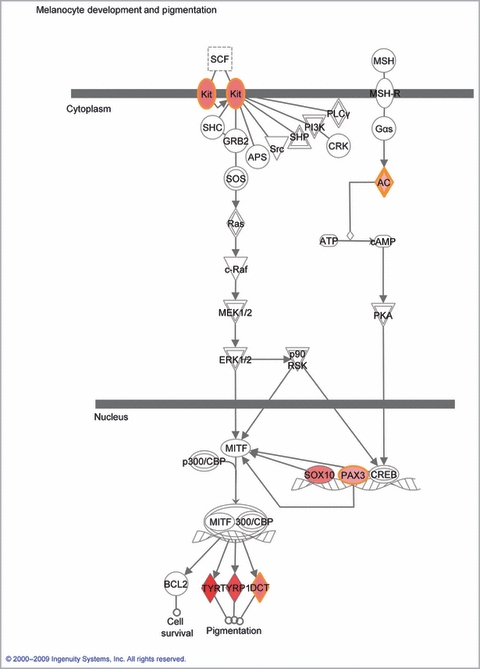
Ingenuity Pathway Analysis of epidermal genetic information retrieval-harvested melanoma specimens identifies overexpressed genes involved in melanocyte development and pigmentation. Melanocyte development and pigmentation is primarily regulated by microphthalmia-associated transcription factor (MITF), which is controlled mainly through the melanocyte-stimulating hormone (MSH) signalling pathway that includes adenylate cyclase 2 (ADCY2), sex determining region Y-box 10 (SOX10) and paired box 3 (PAX3) as well as the v-kit Hardy–Zuckerman 4 feline sarcoma viral oncogene homolog (Kit) signalling pathway. Tyrosinase (TYR), tyrosinase-related protein 1 (TYRP1) and dopachrome tautomerase (DCT) are activated by MITF and are also involved in skin pigmentation.

The biological functions and molecular networks of the 312 genes were assessed by IPA and, interestingly, genes upregulated in the heat map cluster containing melanoma were found to play a role in melanocyte development and pigmentation signalling as well as skin development, cellular development and cancer (Table 2). Some of the genes upregulated in these EGIR-harvested stratum corneum specimens overlying melanoma are shown in Figure 2 including KIT, tyrosinase (TYR), tyrosinase-related protein 1 (TYRP1), dopachrome tautomerase (DCT), SOX10 and PAX3 – all of which are involved in melanocyte development and pigmentation. In addition, other genes known to be expressed in melanocytes, including MLANA, SILV, EDNRB and melanophilin, were also detected in these EGIR specimens overlying melanoma. Some of the genes upregulated in the heat map cluster containing naevi and normal skin are involved in cellular growth and proliferation, embryonic development, and skeletal and muscular system development and function. These findings support the hypothesis that the EGIR method can be used to distinguish melanoma from naevi and normal skin. Further, these observations suggest that the gene expression profile of mRNA found in the stratum corneum overlying a pigmented lesion is altered, either directly or indirectly, by the presence of melanoma.

**Table 2:** Top biological functions of 312 differentially expressed genes among melanoma, naevi and normal skin controls

	No. of molecules	Ratio	*P*-value
Molecular and cellular functions			
Amino acid metabolism	5		2·73 × 10^−8^–1·40 × 10^−2^
Small molecule biochemistry	12		2·73 × 10^−8^–1·40 × 10^−2^
Cellular development	67		5·78 × 10^−8^–1·40 × 10^−2^
Cellular growth and proliferation	66		1·25 × 10^−6^–1·40 × 10^−2^
Cell death	63		1·34 × 10^−6^–1·40 × 10^−2^
Physiological system development and function			
Hair and skin development and function	20		2·73 × 10^−8^–1·40 × 10^−2^
Embryonic development	30		4·99 × 10^−6^–1·40 × 10^−2^
Renal and urological system development and function	13		6·37 × 10^−5^–1·40 × 10^−2^
Organ development	37		7·43 × 10^−5^–1·40 × 10^−2^
Cardiovascular system development and function	35		1·53 × 10^−4^–1·40 × 10^−2^
Diseases and disorders			
Cancer	83		2·32 × 10^−9^–1·40 × 10^−2^
Gastrointestinal disease	33		1·37 × 10^−6^–1·40 × 10^−2^
Skeletal and muscular disorders	74		3·74 × 10^−6^–1·40 × 10^−2^
Infectious disease	33		5·42 × 10^−6^–9·34 × 10^−3^
Respiratory disease	17		5·42 × 10^−6^–2·44 × 10^−3^
Canonical pathways			
Melanocyte development and pigmentation signalling		7/88 (0·08)	1·35 × 10^−4^
Factors producing cardiogenesis in vertebrates		6/89 (0·067)	9·33 × 10^−4^
Axonal guidance signalling		12/403 (0·03)	4·39 × 10^−3^
Human embryonic stem cell pluripotency		6/148 (0·041)	6·46 × 10^−3^
Wnt/β-catenin signalling		7/168 (0·042)	6·97 × 10^−3^

### Identification of a 17-gene classifier to distinguish melanoma from naevi

Having demonstrated that EGIR tape-stripped specimens can be used to detect melanoma, we next asked whether we could develop an approach to predict the risk that a pigmented lesion contains a melanoma. Our strategy was to develop a multigene class predictor (i.e. classifier) to distinguish a melanoma from a naevus using the expression profiles of known samples – a training set consisting of melanoma and naevi – and then use the resultant multigene classifier to assess an independent group of samples – a test set containing melanomas and naevi (details of the analytical schema are provided in Data S1; see Supporting Information).

Accordingly, we employed EGIR to obtain RNA from 202 tape-stripped melanomas and naevi (Table 1), each of which was profiled on the Affymetrix human U133 plus 2.0 GeneChip. The resulting microarray data were randomly divided into training and test datasets. The training set consisted of data from 37 melanomas, including *in situ* (14) and invasive superficial spreading melanomas (19), lentigo maligna (3) and lentigo maligna melanoma (1), and 37 naevi (both dysplastic and nondysplastic), while the test set contained data from 39 melanomas including *in situ* (11) and invasive superficial spreading melanomas (21), lentigo maligna (2), lentigo maligna melanoma (4) and nodular melanoma (1), and 89 naevi (both dysplastic and nondysplastic). The training dataset yielded some 422 genes differentially expressed between melanomas and naevi, as determined by Student's *t*-test with multiple testing correction (*P*<0·05, FDR *q*<0·05) (the annotated list of genes is provided in Table S2; see Supporting Information). The group of 422 differentially expressed genes was then subjected to class prediction modelling to identify a multigene classifier capable of discerning a melanoma from a naevus after which its performance was assessed by the independent, test set data. This was accomplished by stochastic gradient boosting analysis, using the TreeNet data mining algorithm, which permits selection and performance testing of a multigene classifier. Unlike classical modelling techniques, the stochastic gradient boosting algorithm utilizes very flexible structural assumptions and is capable of accommodating multiple sets of genes associated with different classification outcomes in a true multivariate fashion. We found that a classifier from the training set containing 168 of the 422 genes was sufficient to correctly identify all 37 melanomas and 35 of 37 naevi (the annotated list of the 168 genes is provided in Table S3; see Supporting Information). Thus, the sensitivity and specificity of detecting melanoma in the training dataset was 100% and 95%, respectively. The performance of this class prediction model was evaluated with an independent test dataset of 39 melanomas and 89 naevi. All 39 of the melanomas in the test dataset were accurately identified and 78 of the 89 naevi were called correctly by the 168-gene classifier, indicative of 100% sensitivity and 88% specificity. The pathology of the false-positive specimens was variable and included dysplastic naevus (*n*=7), compound naevus (*n*=5) and junctional naevus (*n*=1).

Further TreeNet modelling revealed that the classifier could be reduced to a set of 17 of 168 genes that remained 100% sensitive and 88% specific upon testing (Tables 3 and 4; more complete annotation on these 17 genes is provided in Table S4; see Supporting Information). Furthermore, receiver operating characteristic (ROC) curve analysis of the application of the 17-gene classifier to the test dataset resulted in an area under the curve (AUC) of 0·955, indicating that it is extremely accurate for melanoma detection. We used the 17-gene classifier to evaluate several types of EGIR-harvested additional control lesions, including 73 normal, nonlesional skin samples from both healthy subjects (*n*=53) and patients with melanoma (*n*=19) or basal cell carcinoma (*n*=1), 18 pigmented basal cell carcinomas, and 22 solar lentigines. The assay for melanoma of these specimens was negative for all of the nonlesional skin specimens and the solar lentigines, as well as 17 of the 18 basal cell carcinomas (data not shown). These findings provide further support for the specificity of the 17-gene classifier to detect melanoma specifically.

**Table 3:** A 17-gene melanoma classifier accurately discriminates melanoma from naevi

	Training set		Test set	
		
Histopathological diagnosis	Melanoma	Naevus	Melanoma	Naevus
Melanoma	37	0	39	0
Naevus	2	35	11	78

**Table 4:** List of the 17-gene melanoma classifier

Gene name	GenBank	Description
ACTN4	U48734	Actinin, alpha 4
BC020163	AW342078	Homo sapiens, clone IMAGE:4346533, mRNA
CMIP	AI819630	c-Maf-inducing protein
CNN2	NM_004368	Calponin 2
EDNRB	M74921	Endothelin receptor type B
GPM6B	AW148844	Glycoprotein M6B
KIT	NM_000222	v-kit Hardy–Zuckerman 4 feline sarcoma viral oncogene homolog
MGC40222	N44676	Hypothetical protein MGC40222
NAMPT	NM_005746	Nicotimamide phosphoribosyltransferase
PRAME	NM_006115	Preferentially expressed antigen in melanoma
RPL18	AV738806	Ribosomal protein L18
RPL21	AL356414	Ribosomal protein L21
RPS15	NM_001018	Ribosomal protein S15
TMEM80	AI739035	Transmembrane protein 80
TRIB2	NM_021643	Tribbles homolog 2 (Drosophila)
TTC3	NM_003316	Tetratricopeptide repeat domain 3
VDAC1	AL515918	Voltage-dependent anion channel 1

**Fig 3:**
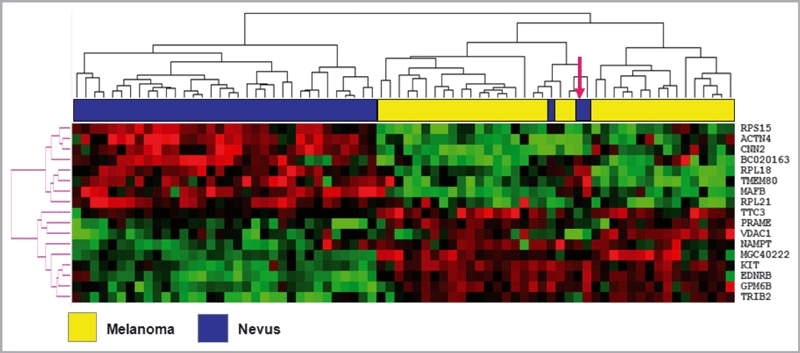
Hierarchical clustering of melanomas and naevi using the 17-gene classifier. Shown are data from the training set of 37 melanomas and 37 naevi. The specimen denoted by the arrow was called a Clark naevus on initial pathological review, but was deemed a melanoma by the 17-gene classifier. The presence of invasive melanoma was detected in this biopsy by both the primary and central dermatopathologists upon re-review of the serially sectioned specimen.

This level of accuracy made us question the original pathology readings on the naevi deemed to be melanomas, or falsely positive, by the 17-gene classifier. Therefore, each of the 13 false-positive specimens was serially sectioned and re-reviewed by both primary and central dermatopathologists. In so doing, it was determined by both pathologists that one of 13 false-positive naevi actually harboured an invasive superficial spreading melanoma (the specimen denoted by an arrow in Fig. 3) (photomicrographs are provided in Fig. S1; see Supporting Information).

### Biological functions of the 17-gene classifier

Among the 17-gene classifier (Table 4), nine genes (ACTN4, CMIP, CNN2, TTC3, VDAC1, NAMPT, RPL21, RPS15 and RPL18) are located in the cytoplasm, five genes (EDNRB, GPM6B, TMEM80, KIT and TRIB2) are in the plasma membrane, PRAME is in the nucleus and two genes (BC020163 and MGC40222) are unknown. KIT, a receptor tyrosine kinase, and EDNRB, a G-protein coupled receptor, are critical regulators in melanocyte development and pigmentation signalling, hair and skin development, and melanoma progression (Fig. 2). The gene encoding preferentially expressed antigen in melanoma (PRAME) is not only involved in melanoma progression but is also implicated in ovarian cancer, neoplasia and breast cancer.^29^,^30^

The biological functions of genes in the 17-gene classifier analysed by IPA were primarily involved in cell death (ACTN4, CNN2, EDNRB, KIT, NAMPT, PRAME, TRIB2 and VDAC1), cellular development (EDNRB, KIT, PRAME, TRIB2, TTC3, CNN2, ACTN4, VDAC1 and NAMPT), hair and skin development (EDNRB and KIT), cancer (EDNRB, KIT, TTC3, NAMPT, PRAME and RPS15) and neurological disease (EDNRB, KIT, NAMPT, RPL21 and TTC3). In addition, EDNRB and KIT were used as clinical drug targets for treatment of metastatic melanoma and so was NAMPT in gastric cancer.^31^–^33^ These results demonstrate that most of the genes in this classifier, which distinguishes melanoma from naevi, are involved in melanoma and cancer.

## Discussion

In this study we have demonstrated that EGIR, noninvasive tape stripping of stratum corneum, can be used to detect melanoma. We have identified 312 genes that are differentially expressed between melanoma, naevi and normal skin. This is the first demonstration that EGIR, a method that samples material from stratum corneum, is capable of detecting signals from cells that typically exist in the basal layer of the epidermis. In addition, EGIR technology was also able to discriminate melanomas from naevi, including dysplastic naevi, with high sensitivity and specificity using 422 differentially expressed genes. Reducing the number of genes to 17, a more practical number that could be tested clinically, still resulted in a classifier with 100% sensitivity and 88% specificity for the detection of *in situ* and invasive superficial spreading melanoma, lentigo maligna and lentigo maligna melanoma with an ROC AUC of better than 0·95. This 17-gene classifier also discriminates melanoma from basal cell carcinoma, solar lentigo and normal skin.

The 17-gene classifier falsely identified 13 naevi as melanoma, raising the question as to why these were misclassified. One possibility is due to a sampling error by histopathological examination of a limited number of sections from the biopsied specimen. This is clearly the case for one of the specimens (denoted by an arrow in Fig. 3), which when serial sectioned and re-reviewed was found to harbour a melanoma. This had been missed during the initial histopathological reading due to the fact that serial sectioning of the biopsied lesion is not routinely performed per standard of care. Another possibility is that EGIR-based genomic assay can detect molecular changes prior to the development of morphological abnormalities in melanoma cells. A third possibility is that these false-positive naevi exhibited pagetoid spread of melanocytes. After re-reviewing the tissue biopsy specimens we could find little, if any, evidence of pagetoid spreading in these false-positive naevi. If these results can be confirmed, it would suggest that the EGIR-based genomic assay may be a more sensitive means to detect melanoma than the standard histopathological review.

Tape stripping removes mainly the stratum corneum, the layer of the skin that does not contain nuclei. The pigment-producing cell, the melanocyte, however, resides in the basal cell layer of the epidermis. Thus, it is surprising that one can isolate mRNA, indicative of this neoplasm, from the skin's horny layer. Several observations may explain this finding. It is known that the distal portion of the melanocyte's dendritic processes are actively phagocytosed by keratinocytes and the melanin contained in these processes is then dispersed throughout the keratinocyte layers, eventually arriving in the stratum corneum.^34^ This appears to be the mechanism by which pigment moves to the upper/outermost layers of the integument from the melanocyte that produces the melanin. Conceivably, the mRNA synthesized by the melanocyte also moves upwards in the epidermis during this process. The keratinocyte may also phagocytose the dendritic processes of Langerhans cells. This would explain why mRNA indicative of an allergic reaction, thought to be a function of the Langerhans cells resident in the suprabasal cell layer, can also be found in the stratum corneum.^21^ In addition, documentation of the mRNA indicative of an abnormal skin reaction at the skin's surface may be explained by the recent observation that the dendritic processes of Langerhans cells penetrate through the tight junctions of the keratinocytes and exit the skin just beneath the stratum corneum where the RNA could be picked up by the tape stripping.^35^

Another possibility for our findings is the phenomenon of cell–cell crosstalk (see below). In this scenario the malignant melanocyte in the epidermis would activate the keratinocytes adjacent to it to generate melanocytic mRNAs, and this process would continue until the abnormal expression of mRNAs would manifest itself in the uppermost layer of the stratum granulosum when the nucleus becomes extruded. From there, the mRNAs indicative of a melanoma could be detected by tape stripping.

Alternatively, the RNA from malignant melanocytes could be sampled by EGIR if the malignant cell is present in the stratum corneum or if tape stripping extends well below into the stratum granulosum. The former is unlikely because it is thought to be a rare event and therefore not capable of generating a signal strong enough to overcome the surrounding noise of cornified keratinocytes. The latter also would not provide a clear explanation, as histopathological review of specimens typically did not show that tape stripping resulted in penetration beyond the stratum corneum layer. However, it is possible that dendritic processes from the malignant melanocytes could extend into the stratum corneum that might be sampled during the process of EGIR.

Recent findings on the genetics of melanoma have shown distinct subsets of the disease in which specific genomic aberrations have been associated with histological subtypes of melanoma, the stage of the disease, and especially with the amount of sun exposure.^36^–^38^ Strikingly, our findings indicate that a core of genes is expressed in both *in situ* and invasive melanoma and in lesions of various histologies, including those associated (i.e. lentigo maligna) or not associated (i.e. superficial spreading) with chronic sun exposure. Among 142 upregulated genes in melanoma, many are known to be expressed specifically in melanocytes, including KIT, Melan-A, TYR, TRPM1, EDNRB, DCT, SOX10 and SILV (Fig. 2). In addition, the biological functions of these upregulated genes are also involved in melanocyte biology, skin/hair pigmentation, cellular growth/proliferation and cancer (Fig. 2, Table 2), thereby suggesting that many are biologically relevant to processes occurring within the underlying epidermal tissue. Thus, it is possible that these genes represent a common thread of genetic progression for melanoma development that occurs at the earliest stage of disease – and may provide a target or group of targets for therapeutic intervention. Lastly, the histopathological differentiation of lentigo maligna and lentigo maligna melanoma from solar lentigo can often be extremely difficult. This 17-gene classifier separates this form of melanoma from the benign process, providing a potential tool for the accurate diagnosis of these difficult pigmented lesions.

Preliminary qRT-PCR results for expression of the 17-gene classifier from 10 melanomas and 10 naevi showed very similar results to the data from the GeneChip microarray, with all 10 melanomas and nine of 10 naevi being discerned correctly (Data S2; see Supporting Information). This preliminary finding strongly suggests that the classifier, discovered by microarray analyses of EGIR specimens, will translate well on to a qRT-PCR platform that should be cost-effective for use in the clinical setting. Should these findings be recapitulated in an expanded clinical validation study, such an assay may be substantially more accurate and certainly more objective than the visual and optical means currently available for melanoma detection. Such a test would also permit clinicians to be vastly more selective in their choice of pigmented skin lesions that need to be biopsied and further evaluated.

**What's already known about this topic?**

- Adhesive tape stripping of skin yields RNA from stratum corneum that can be assayed for gene expression to assess allergic and irritant skin reactions and psoriasis.

**What does this study add?**

- RNA from tape-stripped skin identified genes present in stratum corneum that are differentially expressed between melanoma, naevus and normal skin specimens.
- A 17-gene genomic biomarker was characterized that accurately discriminates *in situ* and invasive melanomas from naevi.

## Supporting Information

**Fig S1.** Photomicrographs of a melanoma, not identified on initial histopathological evaluation, which was detected by the 17-gene melanoma classifier.

**Table S1.** Table of 312 differentially expressed genes between melanomas, naevi and normal skin specimens.

**Table S2.** Table of 422 differentially expressed genes between melanomas and naevi.

**Table S3.** Table of 168 genes identified by the TreeNet analysis in the training set.

**Table S4.** Description of the 17-gene melanoma classifier.

**Data S1.** Details of strategy for melanoma class prediction modelling.

**Data S2.** Assay of melanoma and nonmelanoma specimens by quantitative real-time reverse transcription–polymerase chain reaction using the 17-gene classifier recapitulates microarray results.
