# Supplementary material for: Noninvasive genomic detection of melanoma
Source: Br J Dermatol. 2011 Apr;164(4):797–806. doi: 10.1111/j.1365-2133.2011.10239.x (PMC3118279; doi:10.1111/j.1365-2133.2011.10239.x)
Supplement: Supplementary file 1 [file bjd0164-0797-SD1.pdf]

SUPPORTING INFORMATION

Fig S1. Photomicrographs of a melanoma, not identified on initial histopathological evaluation, which was detected by the 17-gene melanoma classifier

Table S1. Table of 312 differentially expressed genes between melanomas, naevi and normal skin specimens

Table S2. Table of 422 differentially expressed genes between melanomas and naevi

Table S3. Table of 168 genes identified by the TreeNet analysis in the training set

Table S4. Description of the 17-gene melanoma classifier

Data S1. Details of Strategy for Melanoma Class Prediction Modeling

Data S2. Assay of melanoma and nonmelanoma specimens by quantitative real-time reverse transcription-polymerase chain reaction using the 17-gene classifier recapitulates microarray results

Fig S1. Photomicrographs of a melanoma, not identified on initial histopathological evaluation, which was detected by the 17-gene melanoma classifier

The initial histopathologic diagnosis of a mid-lumbar skin lesion (A, B) was a Clark naevus. The 17-gene classifier, however, characterized the EGIR-harvested specimen as a melanoma (see specimen denoted by the arrow in Fig 3). Therefore, serial sectioning of the biopsy was performed and re-reviewed by both the primary and central dermatopathologists. Based on these additional sections (see representative photomicrographs shown in C and D) the pathology of the lesion was revised to malignant melanoma, superficial spreading type, Clark's level II and Breslow thickness 0.37 mm, arising in associated with a compound naevus, with moderate host response.

Photomicrographs of the biopsied mid-lumbar skin lesion: A & B) sections show skin with irregularly nested and single melanocytic naevus cells along the dermal-epidermal junction. Nests bridge between adjacent rete ridges and melanocytic naevus cells are also seen in the dermis. Dermal changes include fibroplasias, mild mononuclear cell inflammation and pigment incontinence. C & D) sections show focal areas of large atypical melanocytes with pleomorphic nuclei, abundant cytoplasm and granular pigment. Single and nested atypical melanocytes are prominent in the lower epidermis with single cells also seen in the mid-epidermis. There are focal areas of atypical epithelioid melanocytes invading downward below the junctional atypia into the papillary dermis, surrounded by a moderate lymphocytic host response.

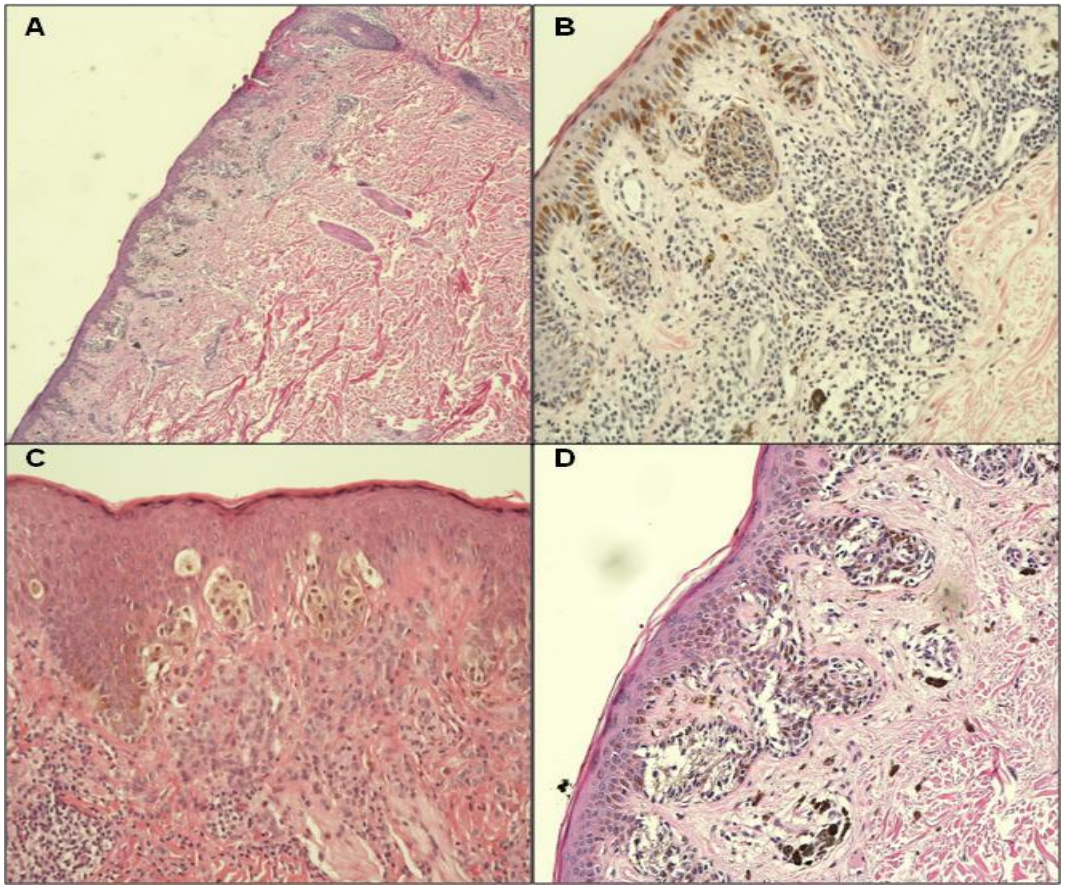

Table S1. Table of 312 differentially expressed genes between melanomas, naevi and normal skin specimens

Probeset ID	Genbank	Gene Symbol	Nevi-mean	Nevi-StdErr	Melanoma-mean	Melanoma-StdErr	NS-mean	NS-StdErr	P-value	Description
1552620_at	NM_173080	SPRR4	3374.40	438.53	518.34	256.22	6451.33	913.77	1.74E-10	small proline rich protein 4
1553613_s_at	NM_001453	FOXC1	961.06	97.19	86.03	19.55	1073.47	162.59	7.66E-13	forkhead box C1
1554821_a_at	BC015030	ZBED1	150.87	18.54	16.07	3.64	82.00	13.77	2.99E-09	zinc finger, BED-type containing 1
1555505_a_at	BC027179	TYR	52.31	24.60	373.62	75.66	6.53	0.19	1.59E-13	tyrosinase (oculocutaneous albinism IA)
1555579_s_at	BC029442	PTPRM	80.84	23.76	964.00	119.98	37.07	11.78	7.49E-17	protein tyrosine phosphatase, receptor type, M
1556182_x_at	BC018095	DRAP1	173.97	18.30	30.90	1.45	83.20	17.50	4.51E-09	Similar to ankyrin
1556590_s_at	T83966		28.09	7.54	91.55	19.65	7.80	0.37	4.35E-09	CDNA FLJ25645 fis, clone SYN00113
1556839_s_at	AA515490	SPTBN5	537.85	89.19	11.59	2.63	880.20	118.01	1.54E-20	Spectrin, beta, non-erythrocytic 5
1557292_a_at	AW665790	MCOLN3	57.63	21.72	218.86	36.68	10.60	0.88	3.73E-16	mucolipin 3
1557797_a_at	AW611486	ZFHX1B	82.84	14.54	1066.03	139.85	88.73	19.31	2.13E-11	Zinc finger homeobox 1b
1559360_at	AL833045	EFNA5	183.37	18.06	22.86	3.75	158.87	28.65	6.87E-10	Nuclear RNA-binding protein, putative
200601_at	U48734	ACTN4	493.62	41.87	89.72	12.78	265.20	54.94	1.91E-09	actinin, alpha 4
200665_s_at	NM_003118	SPARC	119.72	20.64	878.14	113.90	71.60	26.99	1.17E-11	osteonectin
200862_at	NM_014762	DHCR24	178.29	16.94	92.48	25.64	299.13	29.20	5.83E-09	24-dehydrocholesterol reductase
200958_s_at	NM_005625	SDCBP	518.28	113.58	3158.07	308.14	348.80	67.03	5.17E-13	syndecan binding protein (syntenin)
201010_s_at	NM_006472	TXNIP	1080.18	81.14	341.86	50.40	1971.07	232.72	1.33E-09	thioredoxin interacting protein
201147_s_at	BF347089	TIMP3	1488.65	187.32	75.93	19.84	1713.07	307.24	1.66E-16	TIMP metallopeptidase inhibitor 3
201148_s_at	AW338933	TIMP3	292.09	51.61	15.76	3.71	270.20	47.32	3.86E-15	TIMP metallopeptidase inhibitor 3
201149_s_at	U67195	TIMP3	464.91	76.74	13.07	3.40	295.40	45.50	3.78E-18	TIMP metallopeptidase inhibitor 3
201150_s_at	NM_000362	TIMP3	351.13	56.44	21.79	4.00	641.40	118.61	2.18E-17	TIMP metallopeptidase inhibitor 3
201287_s_at	NM_002997	SDC1	1418.13	127.71	258.21	64.26	1087.07	137.03	1.07E-08	syndecan 1
201470_at	NM_004832	GSTO1	389.99	38.78	1174.52	159.35	394.60	33.51	2.51E-10	glutathione S-transferase omega 1
201674_s_at	BC000729	AKAP1	83.54	6.61	16.66	2.34	40.07	6.14	5.98E-10	A kinase (PRKA) anchor protein 1
201745_at	NM_002822	PTK9	89.32	10.40	266.00	27.82	98.07	11.47	4.21E-10	PTK9 protein tyrosine kinase 9
201909_at	NM_001008	RPS4Y1	958.93	116.20	779.93	159.30	1813.13	133.75	1.53E-09	ribosomal protein S4, Y-linked 1
201945_at	NM_002569	FURIN	506.54	47.48	77.07	15.34	229.27	50.04	4.38E-09	furin (paired basic amino acid cleaving enzyme)
202071_at	NM_002999	SDC4	234.88	18.75	43.83	4.85	263.67	58.17	1.23E-10	syndecan 4 (amphiglycan, ryudocan)
202088_at	AI635449	SLC39A6	482.88	45.97	1646.83	155.30	446.93	44.45	4.63E-10	solute carrier family 39, member 6
202158_s_at	NM_006561	CUGBP2	38.87	8.55	272.59	35.45	62.13	11.55	1.4E-09	CUG triplet repeat, RNA binding protein 2
202291_s_at	NM_000900	MGP	109.69	39.90	6.10	0.39	343.53	72.94	9.56E-14	matrix Gla protein
202435_s_at	AU154504	CYP1B1	1088.10	108.21	56.90	22.44	572.00	108.39	7.77E-12	cytochrome P450, family 1, subfamily B
202436_s_at	AU144855	CYP1B1	2006.94	202.45	108.86	53.98	1754.80	339.03	2.85E-12	cytochrome P450, family 1, subfamily B
202437_s_at	NM_000104	CYP1B1	701.65	75.68	45.90	12.21	604.47	156.09	1.19E-09	cytochrome P450, family 1, subfamily B
202478_at	NM_021643	TRIB2	150.46	39.64	967.41	143.80	191.60	29.76	6.84E-12	tribbles homolog 2 (Drosophila)
202479_s_at	BC002637	TRIB2	140.90	38.90	545.97	95.64	65.67	11.10	6.75E-09	tribbles homolog 2 (Drosophila)
202500_at	NM_006736	DNAJB2	842.85	68.67	118.86	16.40	795.60	144.79	6.21E-13	DnaJ (Hsp40) homolog, subfamily B, member 2
202551_s_at	BG546884	CRIM1	1034.71	91.24	131.07	22.50	1105.53	190.61	4.61E-10	cysteine rich transmembrane BMP regulator 1
202592_at	NM_001487	BLOC1S1	337.74	30.97	74.97	8.85	360.27	57.73	5.86E-10	biogenesis of lysosome-related organelles complex-1, subunit 1
202651_at	NM_014873	LPGAT1	37.47	4.24	138.97	17.98	26.93	3.02	1.77E-09	lysophosphatidylglycerol acyltransferase 1

Probeset ID: Probeset ID from Affymetrix U133 plus 2.0 array

GeneBank: GeneBank accession number

Nevi-mean: average expression value of nevi samples

Nevi-StdErr: standard error of nevi samples

Melanoma-mean: average expression value of melanoma samples

Melanoma-StdErr: standard error of melanoma samples

NS-mean: average expression value of normal skin samples

NS-StdErr: standard error of normal skin samples

p-value: ANOVA analysis (p < 0.001, Westfall and Young permutation multi-testing correction, false discovery rate, q<0.05)

Probeset ID	Genbank	Gene Symbol	Nevi-mean	Nevi-StdErr	Melanoma-mean	Melanoma-StdErr	NS-mean	NS-StdErr	P-value	Description
202669_s_at	U16797	EFNB2	146.62	15.80	15.28	3.47	97.27	27.12	2.99E-10	ephrin-B2
202730_s_at	NM_014456	PDCD4	709.93	64.03	151.03	17.99	912.67	146.46	1.24E-10	programmed cell death 4
202731_at	NM_014456	PDCD4	774.31	74.42	152.93	22.64	966.00	168.21	4.95E-09	programmed cell death 4
202826_at	NM_003710	SPINT1	144.24	17.30	26.93	5.69	209.93	48.30	7.17E-09	serine peptidase inhibitor, Kunitz type 1
202935_s_at	AI382146	SOX9	2937.12	241.20	274.14	44.09	3029.73	326.07	1.05E-13	SRY (sex determining region Y)-box 9
202936_s_at	NM_000346	SOX9	2349.93	178.47	210.21	33.50	3580.13	364.89	3.84E-18	SRY (sex determining region Y)-box 9
203011_at	NM_005536	IMPA1	25.87	2.82	113.59	15.20	19.87	1.88	1.24E-10	inositol(myo)-1(or 4)-monophosphatase 1
203217_s_at	NM_003896	ST3GAL5	39.19	12.49	303.45	43.58	18.67	3.72	1.84E-14	ST3 beta-galactoside alpha-2,3-sialyltransferase 5
203329_at	NM_002845	PTPRM	12.54	3.76	106.07	17.50	5.27	0.21	6.85E-15	protein tyrosine phosphatase, receptor type, M
203354_s_at	AW117368	PSD3	196.47	19.99	26.62	3.75	207.33	53.56	7.9E-09	pleckstrin and Sec7 domain containing 3
203455_s_at	NM_002970	SAT	709.43	104.75	4129.59	382.18	902.93	120.18	4.2E-12	spermidine/spermine N1-acetyltransferase
203554_x_at	NM_004219	PTTG1	96.62	15.17	395.69	86.77	55.20	14.13	5.66E-09	pituitary tumor-transforming 1
203603_s_at	NM_014795	ZFHX1B	27.96	6.15	296.52	47.91	17.67	3.50	6.75E-12	zinc finger homeobox 1b
203705_s_at	AI333651	FZD7	483.51	48.95	33.83	7.60	526.53	84.29	7.51E-13	frizzled homolog 7 (Drosophila)
203706_s_at	NM_003507	FZD7	618.82	63.60	33.55	6.64	955.40	190.16	4.32E-13	frizzled homolog 7 (Drosophila)
203712_at	NM_014878	KIAA0020	44.85	3.45	101.17	12.08	31.93	3.18	4.41E-09	KIAA0020
203903_s_at	NM_014799	HEPH	70.76	7.93	7.34	0.63	81.13	16.02	2.24E-12	hephaestin
203921_at	NM_004267	CHST2	1862.44	140.16	75.45	15.37	1740.27	191.92	8.2E-20	carbohydrate (N-acetylglucosamine-6-O) sulfotransferase 2
204252_at	M68520	CDK2	109.99	12.08	384.59	47.32	75.40	5.70	5.88E-10	cyclin-dependent kinase 2
204271_s_at	M74921	EDNRB	340.46	138.61	2621.28	297.11	88.73	34.98	1.49E-17	endothelin receptor type B
204273_at	NM_000115	EDNRB	42.62	14.97	325.45	50.46	6.60	0.41	3.15E-24	endothelin receptor type B
204319_s_at	NM_002925	RGS10	41.90	6.76	154.76	17.80	108.00	31.19	3.56E-09	regulator of G-protein signalling 10
204409_s_at	BC005248	EIF1AY	202.85	28.56	225.59	48.10	448.60	52.86	4.51E-09	eukaryotic translation initiation factor 1A, Y-linked
204415_at	NM_022873	G1P3	60.38	30.06	238.38	37.58	50.27	37.68	3.56E-09	interferon, alpha-inducible protein (clone IFI-6-16)
204457_s_at	NM_002048	GAS1	708.97	75.65	82.97	17.20	732.87	133.40	1.04E-08	growth arrest-specific 1
204466_s_at	BG260394	SNCA	54.04	25.38	417.17	75.46	5.87	0.34	6.06E-19	synuclein, alpha (non A4 component of amyloid precursor)
204527_at	NM_000259	MYO5A	38.99	6.08	174.10	21.00	33.13	5.23	1.72E-11	myosin VA (heavy polypeptide 12, myoxin)
204734_at	NM_002275	KRT15	4911.12	484.80	532.34	132.88	7027.20	820.44	2.71E-13	keratin 15
204995_at	AL567411	CDK5R1	33.53	6.82	176.83	26.04	13.27	1.11	1.18E-13	cyclin-dependent kinase 5, regulatory subunit 1 (p35)
205011_at	NM_014622	LOH11CR2A	139.07	12.45	26.55	4.96	193.27	38.48	1.43E-09	loss of heterozygosity, 11, chromosomal region 2, gene A
205051_s_at	NM_000222	KIT	101.07	32.17	1183.48	150.21	43.47	13.19	7.91E-16	v-kit Hardy-Zuckerman 4 feline sarcoma viral oncogene homolog
205107_s_at	NM_005227	EFNA4	328.15	33.17	25.86	3.87	234.00	44.51	1.01E-12	ephrin-A4
205174_s_at	NM_012413	QPCT	799.03	133.43	2500.97	174.37	1161.87	170.89	3.53E-10	glutaminyl-peptide cyclotransferase (glutaminyl cyclase)
205209_at	BC000254	ACVR1B	599.78	28.25	242.72	28.33	799.80	61.99	5.06E-10	activin A receptor, type IB
205290_s_at	NM_001200	BMP2	482.26	105.40	29.41	6.52	561.93	152.55	4.8E-11	bone morphogenetic protein 2
205337_at	AL139318	DCT	133.53	41.39	1367.31	176.26	50.07	17.69	6.44E-14	dopachrome delta-isomerase, tyrosine-related protein 2
205338_s_at	NM_001922	DCT	245.29	80.38	1695.69	263.64	74.87	31.80	2.48E-10	dopachrome delta-isomerase, tyrosine-related protein 2
205471_s_at	AW772082	DACH1	68.71	7.53	6.24	0.40	23.13	3.91	2.76E-12	dachshund homolog 1 (Drosophila)
205694_at	NM_000550	TYRP1	378.79	112.17	3647.41	299.33	44.73	14.10	1.03E-17	tyrosinase-related protein 1
205730_s_at	NM_014945	ABLIM3	232.87	29.12	20.52	4.13	134.13	28.52	5.5E-09	actin binding LIM protein family, member 3
205816_at	NM_002214	ITGB8	71.00	10.98	10.79	1.20	105.00	16.99	2.9E-10	integrin, beta 8
206140_at	NM_004789	LHX2	3069.24	254.49	139.38	49.00	2048.20	315.25	6.06E-13	LIM homeobox 2
206332_s_at	NM_005531	IFI16	220.75	18.15	856.21	71.82	223.33	33.59	7.01E-12	interferon, gamma-inducible protein 16
206376_at	NM_018057	SLC6A15	14.01	3.99	131.86	26.81	6.13	0.52	6.32E-12	solute carrier family 6, member 15
206426_at	NM_005511	MLANA	94.28	33.13	619.76	83.31	11.80	1.71	7.66E-25	melan-A
206427_s_at	U06654	MLANA	386.21	125.46	3390.59	314.71	84.80	25.06	7.49E-17	melan-A
206453_s_at	NM_016250	NDRG2	4584.88	272.05	963.52	195.15	6539.67	480.70	2.47E-12	NDRG family member 2
206471_s_at	NM_005761	PLXNC1	70.57	13.46	209.76	25.95	29.47	7.91	6.26E-09	plexin C1
206479_at	NM_002420	TRPM1	46.54	18.38	362.24	58.49	8.60	0.64	1.04E-20	transient receptor potential cation channel, subfamily M, member 1
206630_at	NM_000372	TYR	131.46	50.33	1836.52	315.89	8.13	0.95	2.91E-27	tyrosinase (oculocutaneous albinism IA)
206696_at	NM_000273	GPR143	44.46	17.69	366.45	81.70	10.33	1.56	3.73E-16	G protein-coupled receptor 143
206701_x_at	NM_003991	EDNRB	80.60	35.89	512.48	94.92	15.20	4.39	1.43E-16	endothelin receptor type B

Probeset ID	Genbank	Gene Symbol	Nevi-mean	Nevi-StdErr	Melanoma-mean	Melanoma-StdErr	NS-mean	NS-StdErr	P-value	Description
206737_at	NM_004626	WNT11	88.12	21.35	17.24	4.27	136.53	24.37	5.42E-09	wingless-type MMTV integration site family, member 11
207030_s_at	NM_001321	CSRP2	17.46	4.37	234.21	57.20	7.73	0.57	7.85E-15	cysteine and glycine-rich protein 2
207065_at	NM_004693	K6HF	1189.97	159.13	14.41	3.73	1220.60	231.18	1.34E-21	cytokeratin type II
207275_s_at	NM_001995	ACSL1	159.31	17.89	77.66	11.77	308.20	28.01	9.5E-10	acyl-CoA synthetase long-chain family member 1
207279_s_at	NM_016365	NEBL	222.69	19.90	54.83	8.06	282.80	40.41	4.21E-10	nebulette
207469_s_at	NM_003662	PIR	48.82	5.49	187.45	25.37	25.53	3.02	5.92E-11	pirin (iron-binding nuclear protein)
207950_s_at	NM_001149	ANK3	415.65	29.64	91.24	20.12	510.80	63.48	9.74E-09	ankyrin 3, node of Ranvier (ankyrin G)
208073_x_at	NM_003316	TTC3	747.13	45.17	2602.14	258.89	1008.13	121.68	6.17E-09	tetratricopeptide repeat domain 3
208086_s_at	M92650	DMD	109.41	10.42	15.83	2.52	118.40	20.95	8.88E-10	dystrophin (muscular dystrophy, Duchenne and Becker types)
208633_s_at	W61052	MACF1	359.71	27.63	75.86	13.75	223.20	19.58	1E-08	microtubule-actin crosslinking factor 1
208637_x_at	BC003576	ACTN1	238.35	29.02	17.79	3.06	179.73	36.29	4.91E-11	actinin, alpha 1
208645_s_at	AF116710	RPS14	6353.66	463.77	2078.00	514.74	8869.93	1091.85	1.72E-09	ribosomal protein S14
208788_at	AL136939	ELOVL5	28.28	3.89	165.86	22.04	42.33	7.79	1E-11	ELOVL family member 5
208791_at	M25915	CLU	65.35	12.92	8.52	0.39	127.73	25.19	7.65E-10	clusterin
208792_s_at	M25915	CLU	165.97	25.71	18.86	2.81	278.47	58.45	9.65E-10	clusterin
208966_x_at	AF208043	IFI16	333.34	28.87	1362.34	128.62	314.00	47.29	4.52E-12	interferon, gamma-inducible protein 16
209120_at	AL037401	NR2F2	16.76	3.33	175.41	35.07	10.80	1.35	1.77E-09	nuclear receptor subfamily 2, group F, member 2
209167_at	AI419030	GPM6B	152.85	20.48	675.62	88.01	115.20	13.42	8.4E-10	glycoprotein M6B
209173_at	AF088867	AGR2	269.00	66.64	11.76	1.79	350.13	108.38	4.8E-10	anterior gradient 2 homolog (Xenopus laevis)
209230_s_at	AF135266	P8	1058.04	75.53	173.48	30.02	596.53	97.70	1.53E-11	p8 protein (candidate of metastasis 1)
209283_at	AF007162	CRYAB	1843.07	113.44	369.41	74.86	2529.87	195.53	1.47E-10	crystallin, alpha B
209289_at	AI700518	NFIB	586.07	59.62	70.48	10.28	443.00	67.22	4.48E-10	Nuclear factor I/B
209290_s_at	BC001283	NFIB	1262.04	110.18	181.03	24.50	1461.47	216.45	6.29E-13	nuclear factor I/B
209295_at	AF016266	TNFRSF10B	69.12	18.92	610.66	80.45	67.20	23.16	5.54E-12	tumor necrosis factor receptor superfamily, member 10b
209386_at	AI346835	TM4SF1	2357.63	270.73	485.07	107.00	3833.40	520.79	9.36E-10	transmembrane 4 L six family member 1
209387_s_at	M90657	TM4SF1	636.07	96.84	129.83	44.44	1345.80	223.31	8.39E-09	transmembrane 4 L six family member 1
209392_at	L35594	ENPP2	100.51	34.42	719.31	169.10	11.00	1.63	1.54E-12	ectonucleotide pyrophosphatase/phosphodiesterase 2
209487_at	D84109	RBPMS	82.40	11.32	371.28	53.54	18.20	3.69	2.49E-12	RNA binding protein with multiple splicing
209569_x_at	NM_014392	D4S234E	124.10	25.93	376.41	55.57	29.33	7.73	1.38E-12	DNA segment on chromosome 4, 234 expressed sequence
209570_s_at	BC001745	D4S234E	1490.32	263.89	5226.17	564.59	532.87	119.51	2.24E-12	DNA segment on chromosome 4, 234 expressed sequence
209617_s_at	AF035302	CTNND2	135.43	14.33	15.59	1.43	100.07	16.43	3.83E-11	catenin (cadherin-associated protein), delta 2
209842_at	AI367319	SOX10	49.97	15.64	334.97	54.62	7.33	0.94	6.89E-19	SRY (sex determining region Y)-box 10
209848_s_at	U01874	SILV	132.96	43.51	1276.59	253.74	16.67	3.19	9.42E-16	silver homolog (mouse)
209866_s_at	R50822	LPHN3	222.35	20.19	18.86	4.09	159.20	28.17	1.29E-12	latrophilin 3
209867_s_at	AF307080	LPHN3	248.19	28.85	19.66	4.03	152.00	26.44	1.34E-09	latrophilin 3
210064_s_at	NM_006952	UPK1B	83.69	37.11	5.86	0.50	189.07	59.64	2.38E-09	uroplakin 1B
210065_s_at	AB002155	UPK1B	71.57	18.69	7.10	0.24	156.27	45.93	4.49E-10	uroplakin 1B
210086_at	AF039196	HR	450.04	36.41	65.31	10.66	323.80	33.00	4.32E-13	hairless homolog (mouse)
210198_s_at	BC002665	PLP1	36.06	13.49	221.14	31.95	6.47	0.60	1.51E-20	proteolipid protein 1
210495_x_at	AF130095	FN1	7.84	1.80	193.07	95.35	5.00	0.00	8.36E-10	fibronectin 1
210582_s_at	AL117466	LIMK2	97.62	6.95	20.52	3.62	115.53	16.26	6.86E-12	LIM domain kinase 2
210592_s_at	M55580	SAT	566.19	97.55	3606.76	384.33	851.33	124.01	1.08E-12	spermidine/spermine N1-acetyltransferase
210645_s_at	D83077	TTC3	399.31	23.52	1312.21	128.08	487.33	65.89	3.97E-09	tetratricopeptide repeat domain 3
210880_s_at	AB001467	EFS	68.18	6.01	16.97	1.95	66.07	9.21	3.74E-09	embryonal Fyn-associated substrate
210944_s_at	BC003169	CAPN3	249.87	66.03	1112.86	218.75	63.67	14.77	2.69E-10	calpain 3, (p94)
210973_s_at	M63889	FGFR1	96.00	13.24	7.79	2.41	41.87	9.56	4.69E-10	fibroblast growth factor receptor 1
210980_s_at	U47674	ASAH1	301.25	29.49	900.48	80.32	230.20	31.85	3.97E-09	N-acylsphingosine amidohydrolase (acid ceramidase) 1
211105_s_at	U80918	NFATC1	108.37	11.04	14.38	1.79	121.27	16.54	4.78E-13	nuclear factor of activated T-cell cytoplasmic calcineurin-dependent 1
211219_s_at	U11701	LHX2	158.24	16.27	10.48	1.94	98.60	17.75	3.65E-12	LIM homeobox 2
211467_s_at	U70862	NFIB	786.85	79.99	51.66	11.28	627.93	100.35	1.24E-12	nuclear factor I/B
211538_s_at	U56725	HSPA2	1867.90	126.41	456.52	65.52	2058.00	218.50	1.42E-09	heat shock 70kDa protein 2
211596_s_at	AB050468	LRIG1	394.06	44.76	56.17	9.76	387.80	57.49	1.3E-10	leucine-rich repeats and immunoglobulin-like domains 1

Probeset ID	Genbank	Gene Symbol	Nevi-mean	Nevi-StdErr	Melanoma-mean	Melanoma-StdErr	NS-mean	NS-StdErr	P-value	Description
211719_x_at	BC005858	FN1	10.74	3.67	295.62	128.45	5.60	0.53	2.29E-12	fibronectin 1 ; fibronectin 1
212144_at	AL021707	UNC84B	1137.28	153.63	155.79	23.76	781.47	143.83	8.53E-11	unc-84 homolog B (C. elegans)
212274_at	AV705559	LPIN1	205.25	26.96	240.00	53.38	552.33	57.52	5.82E-09	lipin 1
212276_at	D80010	LPIN1	71.31	11.25	84.90	13.41	272.07	42.01	8.37E-09	lipin 1
212382_at	BF433429	TCF4	784.12	91.19	88.72	13.74	467.87	51.10	8.45E-12	Transcription factor 4
212631_at	AI566082	STX7	356.74	33.77	1135.28	133.22	325.87	24.73	2.51E-10	Syntaxin 7
212664_at	AL567012	TUBB4	66.28	30.20	311.45	92.68	5.13	0.09	6.09E-15	tubulin, beta 4
212667_at	AL575922	SPARC	19.71	4.09	145.90	25.98	10.80	1.41	6.8E-09	secreted protein, acidic, cysteine-rich (osteonectin)
212776_s_at	AI978623	OBSL1	94.13	10.26	12.72	2.06	71.60	16.96	6.74E-09	obscurin-like 1
212813_at	AA149644	JAM3	38.09	7.53	106.45	14.92	15.47	2.28	2.69E-10	junctional adhesion molecule 3
212915_at	AL569804	PDZRN3	867.82	104.91	173.14	33.80	1121.00	178.32	6.74E-09	PDZ domain containing RING finger 3
212959_s_at	AK001821	GNPTAB	68.84	9.94	275.07	35.62	59.87	9.52	4.12E-09	N-acetylglucosamine-1-phosphate transferase, alpha and beta subunits
213029_at	BG478428	NFIB	1270.84	117.37	158.10	25.96	1493.60	207.58	3.43E-13	Nuclear factor I/B
213032_at	AI186739	NFIB	3347.57	295.37	474.00	67.40	3639.80	404.63	2.57E-12	Nuclear factor I/B
213033_s_at	AI186739	NFIB	605.16	57.28	64.76	12.20	782.20	131.27	6.14E-12	Nuclear factor I/B
213217_at	AU149572	ADCY2	8.93	1.53	102.41	16.84	5.60	0.36	4.09E-13	adenylate cyclase 2 (brain)
213241_at	AF035307	PLXNC1	42.66	14.69	356.00	59.89	15.20	1.60	2.91E-19	plexin C1
213260_at	AU145890	FOXC1	434.24	45.04	50.69	8.56	652.87	100.92	1.89E-13	Forkhead box C1
213272_s_at	AF070596	LOC57146	104.19	7.92	17.72	2.47	71.60	18.20	1.19E-10	promethin
213456_at	AI927000	SOSTDC1	284.84	37.60	21.14	2.58	372.60	81.64	4.21E-10	sclerostin domain containing 1
213496_at	AW592563	LPPR4	23.74	8.24	89.79	21.72	5.00	0.00	2.9E-10	plasticity related gene 1
213692_s_at	AA904259	VDR	111.99	8.90	22.28	2.87	74.00	13.23	3.94E-09	VDR (vitamin D (1,25- dihydroxyvitamin D3) receptor
213702_x_at	AI934569	ASAH1	582.54	65.53	1943.69	188.46	463.73	71.05	1.22E-09	N-acylsphingosine amidohydrolase (acid ceramidase) 1
213789_at	N58493		188.87	23.32	37.59	6.39	197.93	26.64	5.44E-09	similar to hCG2042049 (LOC100292959), mRNA
213857_s_at	BG230614	CD47	539.99	81.09	1934.66	197.81	511.13	54.51	2.48E-10	CD47 antigen
214036_at	BE464799		484.94	47.65	29.03	2.95	369.60	51.53	3.32E-17	CDNA: FLJ22256 fis, clone HRC02860
214278_s_at	AI358939	NDRG2	580.00	47.18	103.69	20.51	964.47	97.81	9.5E-13	NDRG family member 2
214279_s_at	W74452	NDRG2	454.74	41.70	66.21	19.16	770.00	108.22	2.98E-12	NDRG family member 2
214321_at	BF440025	NOV	10.25	2.80	193.79	43.18	5.47	0.13	5.88E-10	nephroblastoma overexpressed gene
214410_at	N32151	TRPM1	23.51	12.55	149.28	26.70	5.07	0.07	2.06E-11	Transient receptor potential cation channel, subfamily M, member 1
214434_at	AB007877	HSPA12A	17.00	4.87	80.72	13.96	6.67	0.94	1.27E-09	heat shock 70kDa protein 12A
214453_s_at	NM_006417	IFI44	14.28	4.95	271.76	62.19	8.40	2.48	8.6E-13	interferon-induced protein 44
214598_at	AL049977	CLDN8	182.74	21.59	10.07	0.88	298.47	47.47	8.64E-15	claudin 8
214662_at	D26488	WDR43	225.88	20.34	525.41	51.86	163.20	18.43	4.7E-09	WD repeat domain 43
214681_at	AI830490	GK	31.44	7.69	155.97	17.03	37.13	6.94	5.84E-10	glycerol kinase
214721_x_at	AL162074	CDC42EP4	122.54	11.35	14.34	2.44	107.53	19.60	1.01E-12	CDC42 effector protein (Rho GTPase binding) 4
214894_x_at	AK023285	MACF1	498.63	32.93	134.28	17.28	290.33	22.99	9.11E-09	microtubule-actin crosslinking factor 1
215221_at	AK025064	FOXP1	56.57	5.52	10.07	0.77	62.47	12.24	1.64E-09	Forkhead box P1
215222_x_at	AK023406	MACF1	687.91	52.84	147.24	21.72	381.87	41.45	4.64E-10	microtubule-actin crosslinking factor 1
215695_s_at	U94357	GYG2	245.66	80.57	1986.48	340.16	46.00	14.44	2.36E-12	glycogenin 2
216442_x_at	AK026737	FN1	7.07	1.49	167.79	89.37	5.00	0.00	1.19E-09	fibronectin 1
216512_s_at	AL139318	DCT	151.12	47.12	944.28	168.20	38.07	14.11	4.29E-10	dopachrome delta-isomerase, tyrosine-related protein 2
216513_at	AL139318	DCT	43.50	13.10	397.41	62.78	8.53	1.00	5.46E-17	dopachrome delta-isomerase, tyrosine-related protein 2
216874_at	U80770		8.84	1.74	112.69	22.03	5.13	0.09	1.66E-13	Homo sapiens, clone IMAGE:5538654, mRNA
216877_at	U80770		18.32	4.01	195.90	36.27	11.60	3.05	1.83E-11	Homo sapiens, clone IMAGE:5538654, mRNA
217730_at	NM_022152	TMBIM1	557.99	38.41	146.52	19.76	762.93	98.57	2.26E-10	transmembrane BAX inhibitor motif containing 1
217738_at	BF575514	PBEF1	43.03	5.83	207.21	28.80	78.93	11.40	6.14E-10	pre-B-cell colony enhancing factor 1
217739_s_at	NM_005746	PBEF1	98.03	14.05	451.17	54.71	191.67	29.77	1.42E-09	pre-B-cell colony enhancing factor 1
217897_at	NM_022003	FXYD6	514.47	54.48	19.03	3.10	781.13	113.78	6.88E-26	FXYD domain containing ion transport regulator 6
217991_x_at	NM_018070	SSBP3	172.13	17.17	24.97	2.12	50.93	8.49	8.49E-10	single stranded DNA binding protein 3
218062_x_at	NM_012121	CDC42EP4	424.91	36.31	57.72	8.78	276.47	35.94	5.46E-11	CDC42 effector protein (Rho GTPase binding) 4
218196_at	NM_014028	OSTM1	79.50	15.80	425.00	70.92	51.27	5.17	2.99E-10	osteopetrosis associated transmembrane protein 1

Probeset ID	Genbank	Gene Symbol	Nevi-mean	Nevi-StdErr	Melanoma-mean	Melanoma-StdErr	NS-mean	NS-StdErr	P-value	Description
218211_s_at	NM_024101	MLPH	57.47	17.60	834.83	143.64	12.73	2.50	6.06E-19	melanophilin
218236_s_at	NM_005813	PRKD3	27.01	5.87	173.17	27.44	18.00	2.62	5.76E-10	protein kinase D3
218417_s_at	NM_017842	FLJ20489	59.28	8.66	7.21	0.41	36.07	6.63	4E-11	hypothetical protein FLJ20489
218433_at	NM_024594	PANK3	58.21	5.93	17.45	2.45	65.00	8.12	8.36E-10	pantothenate kinase 3
218574_s_at	NM_014583	LMCD1	540.50	54.88	41.10	10.19	542.07	76.08	1.89E-13	LIM and cysteine-rich domains 1
218816_at	NM_018214	LRRC1	256.21	18.36	96.48	13.81	413.33	40.71	1.49E-09	leucine rich repeat containing 1
218966_at	NM_018728	MYO5C	80.85	13.38	13.21	2.58	141.80	21.09	7.01E-10	myosin VC
219142_at	NM_023940	RASL11B	211.06	21.71	20.34	2.35	262.47	44.04	1.42E-13	RAS-like, family 11, member B
219197_s_at	AI424243	SCUBE2	31.46	5.01	151.86	22.37	15.53	2.86	2.06E-12	signal peptide, CUB domain, EGF-like 2
219372_at	NM_014055	CDV1	29.96	5.69	116.24	18.09	19.07	2.49	7.01E-10	carnitine deficiency-associated, expressed in ventricle 1
219412_at	NM_022337	RAB38	63.79	8.62	252.59	32.91	48.47	6.66	9.25E-10	RAB38, member RAS oncogene family
219560_at	NM_024627	FLJ21125	185.04	17.53	45.00	6.56	167.73	23.98	4.51E-09	hypothetical protein FLJ21125
219561_at	NM_016429	COPZ2	605.79	60.44	91.79	10.84	586.40	92.51	2.16E-11	coatomer protein complex, subunit zeta 2
219806_s_at	NM_020179	FN5	63.40	6.19	13.86	1.76	90.27	12.83	3.08E-10	FN5 protein
219842_at	NM_019087	ARFRP2	244.31	31.17	56.38	5.11	194.47	26.73	1.67E-10	ADP-ribosylation factor related protein 2
220178_at	NM_021731	C19orf28	56.76	16.71	319.31	60.75	42.80	13.75	7.61E-10	chromosome 19 open reading frame 28
220911_s_at	NM_025081	KIAA1305	82.49	9.82	15.97	1.91	61.60	11.14	1.23E-09	KIAA1305
221524_s_at	AF272036	RRAGD	20.12	4.94	126.59	19.69	7.93	0.96	2.49E-12	Ras-related GTP binding D
221652_s_at	AF274950	C12orf11	35.76	3.26	104.69	11.52	28.00	4.77	8.06E-09	chromosome 12 open reading frame 11
221664_s_at	AF154005	F11R	115.31	10.10	28.45	3.98	90.27	9.12	3.31E-10	F11 receptor
222153_at	AK023133	MYEF2	20.68	7.77	147.48	28.85	5.07	0.07	7.43E-16	myelin expression factor 2
222294_s_at	AW971415	EIF2C2	116.91	18.70	626.38	80.42	72.20	17.01	4.8E-10	Eukaryotic translation initiation factor 2C, 2
222565_s_at	BF978541	PRKD3	179.35	34.51	580.69	68.27	106.40	22.60	1.65E-09	protein kinase D3
222771_s_at	BF224052	MYEF2	62.24	21.65	407.24	47.30	9.00	1.15	3.06E-26	myelin expression factor 2
223092_at	AA854943	ANKH	263.65	20.32	98.28	18.32	486.20	49.72	1.74E-09	ankylosis, progressive homolog (mouse)
223096_at	AF161469	NOP5/NOP58	92.41	6.29	279.79	27.97	83.20	8.51	2.38E-11	nucleolar protein NOP5/NOP58
223287_s_at	AF146696	FOXP1	299.71	27.33	24.93	3.78	245.87	34.83	7.92E-16	forkhead box P1
223936_s_at	BC005055	FOXP1	52.35	6.11	7.93	0.78	43.00	5.24	2.98E-12	forkhead box P1
223937_at	BC005055	FOXP1	94.10	11.61	10.76	1.92	73.80	13.29	3.35E-10	forkhead box P1
224791_at	AW513835	DDEF1	63.76	9.57	259.07	36.63	121.73	23.48	7.35E-09	development and differentiation enhancing factor 1
224833_at	BE218980	ETS1	50.41	10.44	397.69	48.03	43.47	7.09	1.12E-13	v-ets erythroblastosis virus E26 oncogene homolog 1 (avian)
224837_at	AW080845	FOXP1	487.54	34.56	98.38	9.73	461.40	45.26	1.23E-11	forkhead box P1
224838_at	AK026898	FOXP1	829.82	53.49	192.55	21.88	986.33	99.78	8.36E-10	forkhead box P1
224889_at	BE888885	FOXO3A	858.56	79.53	259.14	36.73	1373.40	149.32	3.74E-09	forkhead box O3A
225137_at	BF111111	FLJ21918	493.06	40.65	104.86	22.99	506.73	70.80	1.06E-09	RNA binding motif protein 35B
225147_at	AL521959	PSCD3	348.19	55.31	1737.86	170.14	266.20	39.87	3.82E-12	pleckstrin homology, Sec7 and coiled-coil domains 3
225175_s_at	AI569503	SLC44A2	497.22	39.35	73.48	15.98	291.67	36.97	1.54E-09	solute carrier family 44, member 2
225520_at	AL133260	MTHFD1L	16.09	3.45	70.48	10.49	7.87	0.60	1.23E-10	methylenetetrahydrofolate dehydrogenase 1-like
225685_at	AI801777	CDC42EP3	67.41	14.01	300.31	44.32	41.20	5.71	3.79E-10	CDC42 effector protein (Rho GTPase binding) 3
225817_at	AB051536	CGNL1	145.37	14.14	13.55	5.28	109.33	26.68	4E-11	cingulin-like 1
225842_at	AK026181		108.38	17.32	932.38	153.42	84.53	29.91	3.12E-10	CDNA clone IMAGE:5531727
225925_s_at	AA044730	USP48	122.93	17.62	444.90	74.22	91.67	33.86	6.16E-09	ubiquitin specific peptidase 48
225991_at	BE644935	TMEM41A	112.74	9.02	31.79	3.63	144.53	15.49	3.41E-11	Transmembrane protein 41A
226281_at	BF059512	DNER	193.96	28.18	20.28	2.36	730.53	132.87	1.31E-15	delta-notch-like EGF repeat-containing transmembrane
226614_s_at	BE856336	C8orf13	460.90	51.24	37.79	7.35	476.13	59.92	7.49E-17	chromosome 8 open reading frame 13
226641_at	AU157224	LOC91526	50.18	8.94	282.41	38.08	37.73	7.46	6.58E-10	Ankyrin repeat domain 44
226711_at	BF590117	HTLF	39.59	5.19	213.38	29.63	52.47	7.82	8.39E-09	human T-cell leukemia virus enhancer factor
226853_at	AU145366	BMP2K	76.43	6.70	226.41	22.27	97.20	20.98	6.77E-10	BMP2 inducible kinase
226988_s_at	AI709055	MYH14	238.76	29.15	37.72	6.10	169.20	31.04	2.55E-09	myosin, heavy polypeptide 14
227099_s_at	AW276078	LOC387763	70.35	12.97	536.38	109.51	50.13	11.04	1E-08	hypothetical LOC387763
227239_at	AV734839	DRCTNNB1A	15.81	2.77	103.76	18.93	10.07	1.28	2.08E-09	down-regulated by Ctnnb1, a
227317_at	AA045042	LMCD1	505.37	50.55	44.14	7.80	572.20	87.00	2.16E-11	LIM and cysteine-rich domains 1

Probeset ID	Genbank	Gene Symbol	Nevi-mean	Nevi-StdErr	Melanoma-mean	Melanoma-StdErr	NS-mean	NS-StdErr	P-value	Description
227396_at	AI631833		80.91	23.47	390.55	54.14	90.40	21.30	1.18E-09	Homo sapiens, clone IMAGE:4454331, mRNA
227405_s_at	AW340311	FZD8	260.51	36.13	18.34	1.60	226.00	50.55	9.6E-11	frizzled homolog 8 (Drosophila)
227761_at	AW235548	MYO5A	157.13	25.72	713.45	68.28	130.80	24.17	1.72E-11	myosin VA (heavy polypeptide 12, myoxin)
227870_at	AB046848	NOPE	8.79	2.71	133.31	33.35	5.27	0.15	6.36E-12	likely ortholog of mouse neighbor of Punc E11
227955_s_at	BE670307		265.15	26.54	22.03	4.47	391.73	59.56	4.32E-13	CDNA: FLJ22256 fis, clone HRC02860
228152_s_at	AK023743	FLJ31033	35.62	9.68	228.17	43.70	21.00	3.19	6.21E-09	hypothetical protein FLJ31033
228176_at	AA534817	EDG3	158.24	25.66	8.62	0.94	116.20	53.60	3.74E-09	endothelial differentiation, sphingolipid G-protein-coupled receptor, 3
228245_s_at	AW594320	OVOS2	150.06	44.86	1631.69	202.81	145.73	62.48	1.09E-11	ovostatin 2
228307_at	AL137580	EMILIN3	52.97	8.48	8.21	0.93	36.33	9.58	1.14E-09	elastin microfibril interfacer 3
228421_s_at	AI740711	EFEMP1	145.00	18.16	8.66	1.17	73.00	26.79	4.91E-09	EGF-containing fibulin-like extracellular matrix protein 1
228462_at	AI928035	IRX2	289.66	24.50	63.34	8.93	316.73	36.52	1.47E-11	iroquois homeobox protein 2
228496_s_at	AW243081	CRIM1	1195.51	106.59	150.90	26.34	1150.00	178.97	9.72E-11	Cysteine rich transmembrane BMP regulator 1 (chordin-like)
228617_at	AA142842	BIRC4BP	26.15	6.59	240.48	49.61	19.47	4.95	3.63E-09	XIAP associated factor-1
228636_at	AL134708	BHLHB5	296.84	31.79	24.69	4.20	385.73	72.46	1.52E-11	basic helix-loop-helix domain containing, class B, 5
228742_at	AA745978		135.07	13.88	9.69	1.17	282.33	41.08	6.54E-18	Full length insert cDNA clone YQ54B06
228862_at	BF509709	FLJ31164	109.31	12.82	23.79	1.72	102.27	16.95	2.4E-10	T-SNARE domain containing 1
228922_at	AI760446	SHF	430.29	58.89	51.38	5.09	316.53	55.81	2.51E-10	Src homology 2 domain containing F
229004_at	AI970797	ADAMTS15	309.18	37.45	7.79	1.27	333.73	56.90	3.64E-20	ADAM metallopeptidase with thrombospondin type 1 motif, 15
229013_at	BF111029		205.31	27.85	31.83	3.88	210.47	38.56	1.48E-09	LOC440282
229518_at	AA531023	FAM46B	610.25	103.96	73.55	14.78	343.40	69.17	1.77E-09	family with sequence similarity 46, member B
229797_at	AI636080	MCOLN3	91.37	35.23	518.66	86.32	8.13	1.90	1.01E-19	mucolipin 3
229844_at	AI699465		43.79	4.88	7.72	0.84	84.33	11.90	2.6E-12	Transcribed locus
230251_at	AA041523	C6orf176	14.16	4.47	70.34	11.10	5.13	0.13	2.39E-09	chromosome 6 open reading frame 176
230264_s_at	AA551090	AP1S2	39.50	8.07	319.62	70.07	27.47	4.77	1.01E-10	adaptor-related protein complex 1, sigma 2 subunit
230285_at	BF447829	DKFZp313A243	103.54	11.71	33.10	5.81	209.47	21.12	2.9E-10	hypothetical protein DKFZp313A2432
230291_s_at	T90642	NFIB	869.94	78.49	94.62	15.87	856.40	144.88	1.15E-10	Nuclear factor I/B
230333_at	BE326919	SAT	57.43	14.52	413.76	77.56	133.87	30.50	1.19E-09	Spermidine/spermine N1-acetyltransferase
230413_s_at	AA480858	AP1S2	19.24	3.90	168.07	35.31	13.53	2.48	2.39E-11	Adaptor-related protein complex 1, sigma 2 subunit
230466_s_at	AI092770		389.63	51.95	1236.59	165.05	223.47	29.56	8.63E-09	Mesenchymal stem cell protein DSC96
230674_at	BE502880	LGR4	67.81	8.06	8.97	0.83	43.40	6.15	7.55E-13	Leucine-rich repeat-containing G protein-coupled receptor 4
230741_at	AI655467		58.56	8.60	486.76	130.40	41.13	7.78	1.72E-09	CDNA FLJ41454 fis, clone BRSTN2011597
231579_s_at	BE968786	TIMP2	901.97	101.77	3492.97	423.55	819.53	89.66	9.56E-10	TIMP metallopeptidase inhibitor 2
231666_at	AA194168	PAX3	17.88	5.31	117.21	17.12	6.13	1.06	6.28E-13	paired box gene 3 (Waardenburg syndrome 1)
232122_s_at	AK022666	VEPH1	16.06	4.53	101.97	17.92	5.87	0.09	1E-10	ventricular zone expressed PH domain homolog 1 (zebrafish)
232217_at	AI292175	C6orf188	300.51	55.99	36.48	14.32	603.40	113.48	1.58E-10	Chromosome 6 open reading frame 188
232504_at	AL389942	LOC285628	21.66	6.38	286.48	47.14	10.27	1.30	8.08E-13	hypothetical protein LOC285628
232602_at	AL050348	WFDC3	1775.94	196.91	50.34	17.25	2653.67	569.73	1.12E-15	WAP four-disulfide core domain 3
232676_x_at	AK002075	MYEF2	89.47	32.85	655.69	68.38	8.20	2.43	5.75E-28	myelin expression factor 2
232983_s_at	AJ243951	DELGEF	126.32	11.89	22.66	8.31	159.93	26.66	4.43E-11	deafness locus associated putative guanine nucleotide exchange factor
233286_at	AK022258	PDZRN3	283.46	35.59	25.55	4.53	189.40	30.19	1.63E-09	PDZ domain containing RING finger 3
233442_at	AU147500	PDZRN3	87.40	9.21	10.28	1.08	39.00	6.60	3.74E-09	PDZ domain containing RING finger 3
233641_s_at	AF124366	C8orf13	405.96	46.64	40.38	9.00	521.87	89.10	4.8E-14	Chromosome 8 open reading frame 13
233814_at	AK025909		1103.22	96.44	98.41	21.86	1551.40	160.33	1.44E-16	CDNA: FLJ22256 fis, clone HRC02860
234120_at	AL117475	LOC439948	653.60	105.90	46.83	5.81	684.67	205.64	1.76E-10	hypothetical gene supported by AL117475
234339_s_at	AF296124	GLTSCR2	219.19	18.26	55.45	2.33	142.93	23.96	3.02E-10	glioma tumor suppressor candidate region gene 2
234351_x_at	AK000948	TRPS1	197.94	19.02	22.83	4.29	199.60	39.77	3.23E-10	trichorhinophalangeal syndrome I
235046_at	AA456099		14.25	3.26	82.24	13.63	6.67	0.48	2.22E-10	Transcribed locus
235182_at	AI816793	C20orf82	636.84	74.42	27.17	6.71	665.33	154.25	1.1E-09	chromosome 20 open reading frame 82
235766_x_at	AA743462	EIF2C2	62.90	9.68	365.38	54.35	43.00	12.31	9.36E-10	Eukaryotic translation initiation factor 2C, 2
236972_at	AI351421	TRIM63	22.59	6.23	440.10	92.60	9.80	1.56	3.59E-15	tripartite motif-containing 63
237069_s_at	AI277662	TRPM1	25.72	10.84	304.31	39.04	5.13	0.13	2.91E-27	transient receptor potential cation channel, subfamily M, member 1
237070_at	AI277662	TRPM1	44.41	12.42	923.34	131.87	10.00	0.14	1.62E-27	transient receptor potential cation channel, subfamily M, member 1

Probeset ID	Genbank	Gene Symbol	Nevi-mean	Nevi-StdErr	Melanoma-mean	Melanoma-StdErr	NS-mean	NS-StdErr	P-value	Description
238362_at	AW271932	PVT1	22.54	3.58	98.14	14.62	10.40	1.45	1.53E-09	Pvt1 oncogene homolog, MYC activator (mouse)
238712_at	BF801735		200.71	18.70	49.76	10.79	290.27	35.31	1.08E-08	Transcribed locus
238756_at	AI860012	GAS2L3	15.82	3.77	135.79	21.85	12.07	2.12	2.71E-10	Growth arrest-specific 2 like 3
239853_at	AI279514	KLC3	92.50	9.13	19.28	3.31	50.00	10.93	2.24E-09	kinesin light chain 3
240386_at	AI224520	TRPM1	24.78	9.41	170.86	35.05	6.53	0.22	8.61E-14	Transient receptor potential cation channel, subfamily M, member 1
241898_at	AA991267		194.21	16.45	45.07	5.49	291.40	32.40	3.18E-10	PREDICTED: similar to KIAA0825 protein
244535_at	AI760944	FOXP1	107.59	10.94	18.48	3.29	108.87	16.85	3.56E-09	Forkhead box P1
244741_s_at	BE855713	MGC9913	339.71	26.77	61.66	11.18	355.20	57.71	8.39E-09	hypothetical protein MGC9913
244829_at	N44676	MGC40222	12.31	2.54	381.76	60.24	6.27	0.23	7.7E-20	Hypothetical protein MGC40222
244845_at	BF725383		330.32	29.20	42.48	5.98	253.20	50.05	1.76E-09	CDNA FLJ45435 fis, clone BRHIP3042817
35254_at	AB007447	TRAFD1	131.01	8.51	44.41	5.47	129.27	17.56	9.1E-09	TRAF-type zinc finger domain containing 1
36030_at	AL080214	HOM-TES-103	28.04	6.64	207.45	47.73	13.60	3.44	7.14E-10	HOM-TES-103 tumor antigen-like
47550_at	N21184	LZTS1	22.62	4.35	121.14	16.17	7.60	1.02	1.27E-14	leucine zipper, putative tumor suppressor 1

Table S2. Table of 422 differentially expressed genes between melanomas and naevi

Probeset	Genbank	Gene Symbol	Nevi-Mean	Nevi-StdErr	Melanoma-Mean	Melanoma-StdErr	p-value	Description
1552652_at	NM_152843	HPS4	80.61	12.16	223.61	35.05	2.23E-04	Hermansky-Pudlak syndrome 4
1555167_s_at	BC020691	PBEF1	143.35	27.54	475.57	66.14	2.11E-05	pre-B-cell colony enhancing factor 1
1555505_a_at	BC027179	TYR	59.07	18.39	359.59	53.14	7.13E-06	tyrosinase (oculocutaneous albinism IA)
1555579_s_at	BC029442	PTPRM	65.71	17.99	430.62	47.44	2.36E-07	protein tyrosine phosphatase, receptor type, M
1556593_s_at	AI192486		66.13	7.92	12.17	1.83	1.67E-04	CDNA FLJ40061 fis, clone TESOP2000083
1556690_s_at	AW341225	ZNF42	79.47	6.69	39.42	3.51	1.73E-04	Zinc finger protein 42 (myeloid-specific retinoic acid-responsive)
1556839_s_at	AA515490	SPTBN5	606.98	73.17	50.91	17.93	2.73E-04	Spectrin, beta, non-erythrocytic 5
1556988_s_at	BE220618	CHD1L	45.24	4.32	82.79	8.32	8.59E-05	chromodomain helicase DNA binding protein 1-like
1557292_a_at	AW665790	MCOLN3	53.73	13.95	203.04	26.96	3.86E-05	mucolipin 3
1557797_a_at	AW611486	ZFHX1B	119.29	32.81	736.29	83.12	3.73E-07	Zinc finger homeobox 1b
1558695_at	AF085937	PLEKHA5	37.95	9.19	119.80	25.03	2.18E-04	Pleckstrin homology domain containing, family A member 5
1559035_a_at	AA115234	AHR	16.28	2.79	29.42	4.46	1.24E-04	Aryl hydrocarbon receptor
1559397_s_at	BE788667	MGC3121	222.18	27.01	64.50	14.35	4.04E-07	hypothetical protein MGC3121
1559759_at	BE407830	KIFC3	33.77	3.75	16.91	1.41	5.30E-05	Kinesin family member C3
200601_at	U48734	ACTN4	507.22	37.69	126.67	14.80	3.05E-07	actinin, alpha 4
200645_at	NM_007278	GABARAP	1207.88	52.96	609.53	68.45	3.50E-05	GABA(A) receptor-associated protein
200665_s_at	NM_003118	SPARC	161.23	24.84	840.08	107.62	1.31E-07	secreted protein, acidic, cysteine-rich (osteonectin)
200747_s_at	NM_006185	NUMA1	117.66	6.55	61.99	4.79	1.67E-04	nuclear mitotic apparatus protein 1
200755_s_at	BF939365	CALU	215.37	18.31	480.09	61.73	9.97E-05	calumenin
200782_at	NM_001154	ANXA5	168.01	24.87	602.76	64.31	3.14E-05	annexin A5
200819_s_at	NM_001018	RPS15	3484.70	171.23	1321.29	108.41	4.04E-07	ribosomal protein S15
200914_x_at	BF589024	KTN1	401.66	22.08	258.91	24.14	5.80E-05	kinectin 1 (kinesin receptor)
200958_s_at	NM_005625	SDCBP	531.24	85.85	2685.15	190.75	3.11E-07	syndecan binding protein (syntenin)
201037_at	NM_002627	PFKP	77.55	7.26	190.99	18.39	2.84E-04	phosphofructokinase, platelet
201091_s_at	BE748755	CBX3	690.74	39.69	1406.55	107.74	4.00E-05	chromobox homolog 3 (HP1 gamma homolog, Drosophila)
201245_s_at	AL523776	OTUB1	253.01	40.00	65.66	8.60	9.45E-05	OTU domain, ubiquitin aldehyde binding 1
201247_at	BE513151	SREBF2	150.01	15.50	45.97	3.85	1.38E-04	sterol regulatory element binding transcription factor 2
201278_at	N21202	DAB2	30.81	5.35	192.20	28.79	7.43E-05	Disabled homolog 2, mitogen-responsive phosphoprotein
201299_s_at	NM_018221	MOBK1B	663.01	27.96	397.14	33.55	2.04E-04	MOB1, Mps One Binder kinase activator-like 1B (yeast)
201423_s_at	AL037208	CUL4A	67.56	4.63	92.51	8.24	1.74E-04	cullin 4A
201428_at	NM_001305	CLDN4	394.44	45.86	108.01	13.81	1.57E-04	claudin 4
201470_at	NM_004832	GSTO1	330.19	26.44	820.41	79.07	1.98E-04	glutathione S-transferase omega 1
201556_s_at	BC002737	VAMP2	816.64	46.55	374.33	37.79	2.46E-06	vesicle-associated membrane protein 2 (synaptobrevin 2)
201565_s_at	NM_002166	ID2	302.26	41.96	63.72	9.93	6.36E-05	inhibitor of DNA binding 2
201603_at	AI817061	PPP1R12A	243.40	16.62	388.04	25.47	3.85E-05	protein phosphatase 1, regulatory (inhibitor) subunit 12A
201605_x_at	NM_004368	CNN2	335.60	19.03	173.58	10.47	1.51E-05	calponin 2
201674_s_at	BC000729	AKAP1	63.69	5.90	18.03	3.83	1.68E-04	A kinase (PRKA) anchor protein 1
201739_at	NM_005627	SGK	2806.10	150.55	5196.49	323.88	1.23E-04	serum/glucocorticoid regulated kinase
201745_at	NM_002822	PTK9	93.82	7.82	202.86	20.72	3.29E-05	PTK9 protein tyrosine kinase 9
201792_at	NM_001129	AEBP1	593.99	98.78	2247.34	259.27	8.60E-05	AE binding protein 1

Probeset ID: Probeset ID from Affymetrix U133 plus 2.0 array

GeneBank: GeneBank accession number

Nevi-mean: average expression value of nevi samples

Nevi-StdErr: standard error of nevi samples

Melanoma-mean: average expression value of melanoma samples

Melanoma-StdErr: standard error of melanoma samples

p-value: t-test ($p < 0.05$, Westfall and Young permutation multi-testing correction, false discovery rate, $q<0.05$)

Probeset	Genbank	Gene Symbol	Nevi-Mean	Nevi-StdErr	Melanoma-Mean	Melanoma-StdErr	p-value	Description
201945_at	NM_002569	FURIN	534.63	53.88	197.37	32.99	4.31E-05	furin (paired basic amino acid cleaving enzyme)
201976_s_at	NM_012334	MYO10	548.20	30.76	1083.45	83.87	1.54E-04	myosin X
202088_at	AI635449	SLC39A6	433.84	35.97	1152.25	84.20	6.31E-05	solute carrier family 39 (zinc transporter), member 6
202142_at	BC003090	COPS8	35.76	5.40	83.18	9.09	1.56E-04	COP9 constitutive photomorphogenic homolog subunit 8
202158_s_at	NM_006561	CUGBP2	44.46	9.61	176.72	19.61	7.89E-05	CUG triplet repeat, RNA binding protein 2
202249_s_at	AU146233	WDR42A	235.82	14.36	163.01	21.45	2.29E-04	WD repeat domain 42A
202307_s_at	NM_000593	TAP1	73.46	5.40	161.66	18.18	2.08E-04	transporter 1, ATP-binding cassette, sub-family B (MDR/TAP)
202370_s_at	NM_001755	CBFB	144.37	13.05	198.16	20.44	9.97E-05	core-binding factor, beta subunit
202426_s_at	BE675800	RXRA	88.17	8.14	37.36	2.63	1.21E-05	retinoid X receptor, alpha
202450_s_at	NM_000396	CTSK	72.84	9.69	286.30	43.78	4.40E-06	cathepsin K (pycnodysostosis)
202478_at	NM_021643	TRIB2	181.01	49.12	888.22	91.35	2.22E-08	tribbles homolog 2 (Drosophila)
202479_s_at	BC002637	TRIB2	120.79	28.52	531.08	66.94	1.54E-04	tribbles homolog 2 (Drosophila)
202571_s_at	BE550798	DLGAP4	269.98	22.86	103.99	20.30	9.03E-06	discs, large (Drosophila) homolog-associated protein 4
202572_s_at	NM_014902	DLGAP4	386.81	26.15	220.17	26.80	4.89E-05	discs, large (Drosophila) homolog-associated protein 4
202589_at	NM_001071	TYMS	45.64	5.55	101.47	15.82	5.65E-05	thymidylate synthetase
202609_at	NM_004447	EPS8	33.75	7.37	115.18	18.65	4.63E-05	epidermal growth factor receptor pathway substrate 8
202651_at	NM_014873	LPGAT1	37.30	3.75	106.76	10.67	1.88E-04	lysophosphatidylglycerol acyltransferase 1
202723_s_at	AW117498	FOXO1A	43.10	4.27	13.53	2.24	2.14E-04	forkhead box O1A (rhabdomyosarcoma)
202734_at	NM_004240	TRIP10	136.98	16.29	64.24	10.59	7.96E-05	thyroid hormone receptor interactor 10
202755_s_at	AI354864	GPC1	65.37	5.47	34.32	2.00	9.97E-05	glypican 1
202879_s_at	AI798823	PSCD1	73.03	6.51	42.88	5.78	1.98E-04	pleckstrin homology, Sec7 and coiled-coil domains 1
202984_s_at	AA457021	BAG5	101.97	6.07	49.21	4.78	2.90E-05	BCL2-associated athanogene 5
203004_s_at	NM_005920	MEF2D	24.90	3.15	12.75	2.12	2.69E-04	MADS box transcription enhancer factor 2, polypeptide D
203011_at	NM_005536	IMPA1	23.41	2.30	69.74	8.39	1.36E-04	inositol(myo)-1(or 4)-monophosphatase 1
203217_s_at	NM_003896	ST3GAL5	47.73	11.12	253.50	31.94	1.78E-05	ST3 beta-galactoside alpha-2,3-sialyltransferase 5
203329_at	NM_002845	PTPRM	13.51	3.12	67.84	8.55	8.41E-05	protein tyrosine phosphatase, receptor type, M
203455_s_at	NM_002970	SAT	867.30	117.46	3602.58	239.90	3.59E-05	spermidine/spermine N1-acetyltransferase
203603_s_at	NM_014795	ZFHX1B	61.21	15.90	311.12	35.03	8.53E-06	zinc finger homeobox 1b
203735_x_at	N35896	PPFIBP1	1051.06	53.76	720.76	25.77	8.66E-06	PTPRF interacting protein, binding protein 1 (liprin beta 1)
203753_at	NM_003199	TCF4	378.50	39.02	100.30	17.70	9.97E-05	transcription factor 4
203842_s_at	NM_012326	MAPRE3	138.29	15.89	34.99	4.07	9.97E-05	microtubule-associated protein, RP/EB family, member 3
203875_at	NM_003069	SMARCA1	20.33	2.31	51.92	5.79	2.32E-04	SMARCA1
204014_at	NM_001394	DUSP4	104.15	35.60	604.41	111.04	9.18E-05	dual specificity phosphatase 4
204040_at	NM_014746	RNF144	18.61	2.14	67.20	8.33	1.23E-04	ring finger protein 144
204086_at	NM_006115	PRAME	7.54	1.80	40.93	6.11	1.44E-06	preferentially expressed antigen in melanoma
204131_s_at	N25732	FOXO3A	897.07	60.13	410.22	47.56	1.37E-04	forkhead box O3A
204252_at	M68520	CDK2	95.42	8.68	286.46	24.74	1.04E-04	cyclin-dependent kinase 2
204271_s_at	M74921	EDNRB	385.40	106.32	2757.37	286.01	1.25E-09	endothelin receptor type B
204273_at	NM_000115	EDNRB	34.53	9.26	252.46	37.20	1.47E-05	endothelin receptor type B
204415_at	NM_022873	G1P3	55.39	20.85	277.26	46.54	6.30E-05	interferon, alpha-inducible protein (clone IFI-6-16)
204466_s_at	BG260394	SNCA	57.67	18.00	312.95	39.29	1.67E-06	synuclein, alpha (non A4 component of amyloid precursor)
204470_at	NM_001511	CXCL1	29.15	15.59	326.34	60.65	3.33E-06	chemokine (C-X-C motif) ligand 1
204527_at	NM_000259	MYO5A	43.13	6.08	141.26	15.81	4.04E-07	myosin VA (heavy polypeptide 12, myoxin)
205051_s_at	NM_000222	KIT	111.95	24.27	926.32	91.49	8.58E-09	v-kit Hardy-Zuckerman 4 feline sarcoma viral oncogene homolog
205260_s_at	NM_001107	ACYP1	87.40	13.65	126.82	16.15	4.51E-05	acylphosphatase 1, erythrocyte (common) type
205337_at	AL139318	DCT	136.26	31.55	904.21	95.61	1.97E-07	dopachrome delta-isomerase, tyrosine-related protein 2
205338_s_at	NM_001922	DCT	289.43	78.08	1375.54	144.57	8.48E-06	dopachrome delta-isomerase, tyrosine-related protein 2
205471_s_at	AW772082	DACH1	59.96	5.66	12.51	3.03	2.13E-04	dachshund homolog 1 (Drosophila)
205681_at	NM_004049	BCL2A1	23.59	6.12	191.78	28.24	1.56E-05	BCL2-related protein A1
205694_at	NM_000550	TYRP1	453.71	96.83	3032.08	198.23	1.69E-07	tyrosinase-related protein 1
205709_s_at	NM_001263	CDS1	157.24	20.18	44.38	10.32	2.17E-05	CDP-diacylglycerol synthase 1

Probeset	Genbank	Gene Symbol	Nevi-Mean	Nevi-StdErr	Melanoma-Mean	Melanoma-StdErr	p-value	Description
205853_at	NM_015872	ZBTB7B	23.55	1.34	15.92	0.81	2.06E-05	zinc finger and BTB domain containing 7B
206180_x_at	NM_023931	MGC2474	374.29	27.72	203.26	10.60	2.17E-05	hypothetical protein MGC2474
206332_s_at	NM_005531	IFI16	209.44	13.79	636.64	53.96	3.72E-05	interferon, gamma-inducible protein 16
206376_at	NM_018057	SLC6A15	15.83	3.47	118.09	16.25	2.51E-06	solute carrier family 6, member 15
206426_at	NM_005511	MLANA	74.63	19.47	390.86	38.09	5.28E-06	melan-A
206427_s_at	U06654	MLANA	454.30	105.95	2958.89	232.89	5.97E-08	melan-A
206479_at	NM_002420	TRPM1	31.94	9.24	185.18	26.35	5.26E-05	transient receptor potential cation channel, subfamily M, member 1
206630_at	NM_000372	TYR	161.63	44.47	1557.17	190.41	5.97E-08	tyrosinase (oculocutaneous albinism IA)
206665_s_at	NM_001191	BCL2L1	815.91	87.49	180.28	27.57	1.23E-04	BCL2-like 1
206696_at	NM_000273	GPR143	42.45	10.84	291.54	43.33	3.70E-06	G protein-coupled receptor 143
206701_x_at	NM_003991	EDNRB	97.32	30.82	622.88	90.02	1.06E-08	endothelin receptor type B
206898_at	NM_021153	CDH19	12.12	2.35	74.21	14.60	2.23E-04	cadherin 19, type 2
207469_s_at	NM_003662	PIR	44.59	5.03	169.82	29.74	2.73E-04	pirin (iron-binding nuclear protein)
207826_s_at	NM_002167	ID3	42.38	2.73	18.00	1.05	1.37E-05	inhibitor of DNA binding 3, dominant negative helix-loop-helix protein
207827_x_at	L36675	SNCA	429.31	45.75	1629.97	163.96	5.99E-06	synuclein, alpha (non A4 component of amyloid precursor)
208073_x_at	NM_003316	TTC3	829.04	46.89	2365.96	180.22	3.50E-05	tetratricopeptide repeat domain 3
208079_s_at	NM_003158	STK6	101.06	25.23	281.26	43.91	2.73E-04	serine/threonine kinase 6
208089_s_at	NM_030794	TDRD3	43.53	5.10	147.71	21.47	1.54E-04	tudor domain containing 3 ; tudor domain containing 3
208738_x_at	AK024823	SUMO2	1255.22	48.09	958.83	63.14	1.26E-05	SMT3 suppressor of mif two 3 homolog 2 (yeast)
208750_s_at	AA580004	ARF1	512.13	36.91	244.50	31.96	1.64E-04	ADP-ribosylation factor 1
208788_at	AL136939	ELOVL5	36.72	4.50	149.99	18.56	1.58E-05	ELOVL family member 5
208966_x_at	AF208043	IFI16	312.66	22.16	1029.38	87.75	2.00E-05	interferon, gamma-inducible protein 16
209000_s_at	BC001329	SEPT8	400.44	31.30	204.32	28.57	3.45E-05	septin 8
209002_s_at	BC003177	CALCOCO1	776.17	55.24	382.96	53.35	1.88E-04	calcium binding and coiled-coil domain 1
209023_s_at	BC001765	STAG2	225.77	14.59	139.32	11.13	2.19E-04	stromal antigen 2
209058_at	AB002282	EDF1	1145.69	68.22	404.32	38.59	3.50E-05	endothelial differentiation-related factor 1
209120_at	AL037401	NR2F2	17.88	2.46	169.79	29.27	1.05E-06	nuclear receptor subfamily 2, group F, member 2
209135_at	AF289489	ASPH	349.00	22.97	738.91	52.33	9.58E-05	aspartate beta-hydroxylase
209168_at	AW148844	GPM6B	50.25	7.43	175.28	18.56	1.69E-07	glycoprotein M6B
209172_s_at	U30872	CENPF	18.50	2.90	55.09	9.94	1.88E-04	centromere protein F, 350/400ka (mitosin)
209199_s_at	N22468	MEF2C	7.11	0.92	37.01	7.09	1.20E-04	MADS box transcription enhancer factor 2, polypeptide C
209204_at	AI824831	LMO4	36.37	3.82	12.63	0.51	2.70E-04	LIM domain only 4
209230_s_at	AF135266	P8	968.08	58.00	251.42	29.81	1.24E-04	p8 protein (candidate of metastasis 1)
209234_at	BF939474	KIF1B	752.31	42.75	394.00	38.98	4.18E-05	kinesin family member 1B
209238_at	BE966922	STX3A	58.81	6.06	90.71	10.26	2.32E-06	syntaxin 3A
209241_x_at	AB041926	MINK1	35.94	2.28	23.30	1.93	9.97E-05	misshapen-like kinase 1 (zebrafish)
209295_at	AF016266	TNFRSF10B	118.02	27.34	567.39	65.81	2.54E-05	tumor necrosis factor receptor superfamily, member 10b
209392_at	L35594	ENPP2	126.37	35.26	657.99	103.50	1.73E-05	ectonucleotide pyrophosphatase/phosphodiesterase 2
209442_x_at	AL136710	ANK3	403.32	26.87	118.13	15.52	1.04E-04	ankyrin 3, node of Ranvier (ankyrin G)
209514_s_at	BE502030	RAB27A	90.54	10.86	410.18	50.07	2.00E-05	RAB27A, member RAS oncogene family
209515_s_at	U38654	RAB27A	51.79	7.74	156.80	17.09	3.72E-05	RAB27A, member RAS oncogene family
209686_at	BC001766	S100B	61.84	18.46	548.08	100.81	4.31E-05	S100 calcium binding protein, beta (neural)
209780_at	AL136883	PHTF2	374.25	24.36	743.29	49.95	2.70E-04	putative homeodomain transcription factor 2
209814_at	BC004421	ZNF330	349.02	28.65	703.05	50.98	1.24E-04	zinc finger protein 330
209842_at	AI367319	SOX10	46.22	11.63	286.76	34.84	5.80E-05	SRY (sex determining region Y)-box 10
209848_s_at	U01874	SILV	246.05	58.54	1673.61	193.76	3.05E-07	silver homolog (mouse)
209867_s_at	AF307080	LPHN3	166.58	17.41	29.24	5.30	1.36E-04	latrophilin 3
209953_s_at	U63131	CDC37	61.43	5.73	32.46	3.70	5.99E-06	CDC37 cell division cycle 37 homolog (S. cerevisiae)
210020_x_at	M58026	CALML3	1841.79	196.75	326.59	47.71	1.90E-04	calmodulin-like 3
210086_at	AF039196	HR	478.98	40.67	94.45	12.33	8.99E-05	hairless homolog (mouse)
210139_s_at	L03203	PMP22	68.16	8.33	228.00	26.84	7.66E-05	peripheral myelin protein 22

Probeset	Genbank	Gene Symbol	Nevi-Mean	Nevi-StdErr	Melanoma-Mean	Melanoma-StdErr	p-value	Description
210198_s_at	BC002665	PLP1	40.67	11.55	233.17	28.92	3.73E-07	proteolipid protein 1
210495_x_at	AF130095	FN1	11.46	2.90	132.12	40.65	3.65E-05	fibronectin 1
210519_s_at	BC000906	NQO1	13.23	2.27	44.34	7.68	9.97E-05	NAD(P)H dehydrogenase, quinone 1
210592_s_at	M55580	SAT	885.24	140.83	3578.58	281.98	1.21E-05	spermidine/spermine N1-acetyltransferase
210645_s_at	D83077	TTC3	425.73	24.98	1167.96	92.66	1.60E-04	tetratricopeptide repeat domain 3
210852_s_at	AF229180	AASS	35.01	3.17	91.87	11.78	1.29E-05	aminoadipate-semialdehyde synthase
210951_x_at	AF125393	RAB27A	86.21	10.75	400.09	44.52	4.15E-05	RAB27A, member RAS oncogene family
210958_s_at	BC003646	MAST4	179.96	14.38	71.63	6.00	2.66E-04	microtubule associated serine/threonine kinase family member 4
211075_s_at	Z25521	CD47	87.38	11.66	213.20	31.74	1.56E-04	CD47 antigen
211084_x_at	Z25429	PRKD3	69.08	12.36	197.63	23.77	4.90E-05	protein kinase D3 ; protein kinase D3
211340_s_at	M28882	MCAM	18.83	3.55	44.03	6.47	2.81E-04	melanoma cell adhesion molecule
211347_at	AF064105	CDC14B	100.33	9.39	310.91	35.14	1.02E-05	CDC14 cell division cycle 14 homolog B (S. cerevisiae)
211348_s_at	AF064105	CDC14B	203.94	16.16	550.28	52.39	2.04E-05	CDC14 cell division cycle 14 homolog B (S. cerevisiae)
211388_s_at	U80761		325.06	23.06	141.78	23.32	4.31E-05	Homo sapiens CTG26 alternate open reading frame mRNA, complete cd
211538_s_at	U56725	HSPA2	1545.89	82.00	507.86	53.77	9.97E-05	heat shock 70kDa protein 2
211546_x_at	L36674	SNCA	78.25	9.01	293.34	30.20	7.11E-06	synuclein, alpha (non A4 component of amyloid precursor)
211622_s_at	M33384	ARF3	545.17	34.16	265.17	28.20	2.61E-04	ADP-ribosylation factor 3 ; ADP-ribosylation factor 3
211719_x_at	BC005858	FN1	14.76	3.92	207.28	58.36	2.02E-05	fibronectin 1 ; fibronectin 1
211905_s_at	AF011375	ITGB4	95.72	15.39	35.32	3.47	2.02E-04	integrin, beta 4
211948_x_at	BG261071	BAT2D1	212.80	13.59	366.41	31.34	4.39E-06	BAT2 domain containing 1
212038_s_at	AL515918	VDAC1	963.19	52.79	1257.05	81.82	1.26E-05	voltage-dependent anion channel 1
212110_at	D31887	SLC39A14	22.98	2.67	48.82	6.98	2.61E-04	solute carrier family 39 (zinc transporter), member 14
212122_at	AW771590	RHOQ	12.92	1.20	40.17	6.02	2.39E-04	ras homolog gene family, member Q ; similar to ARHQ protein
212138_at	AK021757	SCC-112	104.48	6.84	165.96	15.20	8.66E-06	SCC-112 protein
212144_at	AL021707	UNC84B	1278.30	129.59	340.86	55.66	1.74E-04	unc-84 homolog B (C. elegans)
212382_at	BF433429	TCF4	575.38	52.61	100.45	18.44	6.39E-05	Transcription factor 4
212464_s_at	X02761	FN1	11.96	3.14	175.95	56.89	6.56E-05	fibronectin 1
212632_at	N32035	STX7	133.55	14.22	336.21	32.66	1.37E-05	Syntaxin 7
212664_at	AL567012	TUBB4	58.38	19.25	391.99	75.04	4.71E-05	tubulin, beta 4
212667_at	AL575922	SPARC	20.06	3.63	116.04	20.29	1.57E-04	secreted protein, acidic, cysteine-rich (osteonectin)
212672_at	U82828	ATM	24.34	3.01	89.62	12.44	1.20E-04	ataxia telangiectasia mutated
212788_x_at	BG537190	FTL	753.73	64.06	1733.34	193.96	1.45E-04	ferritin, light polypeptide
212810_s_at	W72527	SLC1A4	257.48	42.53	884.99	116.63	2.46E-05	solute carrier family 1, member 4
212820_at	AB020663	DMXL2	27.25	3.79	138.39	22.41	1.87E-04	Dmx-like 2
212829_at	BE878277		21.90	2.88	89.63	14.05	8.60E-05	CDNA FLJ13267 fis, clone OVARC1000964
212959_s_at	AK001821	GNPTAB	44.61	4.86	179.07	20.64	2.46E-05	N-acetylglucosamine-1-phosphate transferase, alpha and beta subunits
212969_x_at	BE222618	EML3	107.85	5.82	70.20	4.38	1.74E-04	echinoderm microtubule associated protein like 3
213007_at	W74442	FLJ10719	27.73	3.53	53.46	5.70	5.21E-05	hypothetical protein FLJ10719
213035_at	AI081194	ANKRD28	9.27	0.77	44.76	6.60	5.60E-05	ankyrin repeat domain 28
213092_x_at	AW241779	DNAJC9	26.91	1.69	48.05	4.10	2.01E-04	DnaJ (Hsp40) homolog, subfamily C, member 9
213119_at	AW058600	SLC36A1	110.04	6.95	168.00	11.54	1.14E-05	solute carrier family 36 (proton/amino acid symporter), member 1
213133_s_at	AW237404	GCSH	169.97	14.11	229.71	18.66	1.67E-04	glycine cleavage system protein H (aminomethyl carrier)
213146_at	AA521267	JMJD3	231.66	13.18	102.86	8.34	4.90E-05	jumonji domain containing 3
213217_at	AU149572	ADCY2	7.91	0.88	58.75	8.34	2.54E-05	adenylate cyclase 2 (brain)
213241_at	AF035307	PLXNC1	60.63	15.24	313.16	37.61	3.35E-05	plexin C1
213272_s_at	AF070596	LOC57146	65.47	4.85	15.82	1.95	2.70E-05	promethin
213361_at	AW129593	TDRD7	45.49	7.26	143.22	21.59	2.46E-04	tudor domain containing 7
213517_at	AW103422	PCBP2	117.60	8.51	55.16	4.96	2.08E-04	Poly(rC) binding protein 2
213688_at	N25325	CALM1	205.48	14.33	88.78	10.39	1.72E-04	calmodulin 1 (phosphorylase kinase, delta)
213755_s_at	BF431501	SKI	112.40	11.96	53.14	7.92	2.23E-04	(v-ski sarcoma viral oncogene homolog
213774_s_at	AW614578	PPP1R2	375.02	62.56	115.75	34.44	2.08E-04	protein phosphatase 1, regulatory (inhibitor) subunit 2

Probeset	Genbank	Gene Symbol	Nevi-Mean	Nevi-StdErr	Melanoma-Mean	Melanoma-StdErr	p-value	Description
213813_x_at	AI345238	FTL	1274.39	105.25	3731.65	401.91	1.15E-04	Ferritin, light polypeptide
213862_at	AI979087	PNPLA2	99.62	14.97	55.80	12.72	5.27E-05	Patatin-like phospholipase domain containing 2
213881_x_at	AI971724	SUMO2	1493.94	61.76	1141.59	74.20	1.01E-04	SMT3 suppressor of mif two 3 homolog 2 (yeast)
213988_s_at	BE971383	SAT	105.26	32.78	446.30	60.72	1.48E-04	spermidine/spermine N1-acetyltransferase
214321_at	BF440025	NOV	11.18	3.33	220.99	97.44	8.82E-05	nephroblastoma overexpressed gene
214410_at	N32151	TRPM1	20.40	7.27	108.54	14.79	1.31E-04	Transient receptor potential cation channel, subfamily M, member 1
214453_s_at	NM_006417	IFI44	18.46	7.15	190.74	32.59	2.10E-05	interferon-induced protein 44
214469_at	NM_021052	HIST1H2AE	522.82	52.34	186.08	26.62	2.47E-04	histone 1, H2ae
214681_at	AI830490	GK	49.40	9.48	171.99	18.10	1.89E-06	glycerol kinase
214792_x_at	AI955119	VAMP2	871.59	53.49	426.61	32.77	1.24E-06	vesicle-associated membrane protein 2 (synaptobrevin 2)
214807_at	AI278204		527.49	32.99	220.72	29.15	7.34E-05	MRNA; cDNA DKFZp564O0862 (from clone DKFZp564O0862)
214894_x_at	AK023285	MACF1	426.01	26.33	122.61	10.21	1.29E-04	microtubule-actin crosslinking factor 1
214925_s_at	AK026484	SPTAN1	433.67	30.79	176.01	17.48	2.36E-04	spectrin, alpha, non-erythrocytic 1 (alpha-fodrin)
215222_x_at	AK023406	MACF1	443.02	30.31	113.88	11.83	1.24E-04	microtubule-actin crosslinking factor 1
215695_s_at	U94357	GYG2	251.79	67.89	1687.61	204.46	5.51E-06	glycogenin 2
215904_at	AL049698	MLLT4	255.92	27.14	85.72	15.70	1.48E-04	myeloid/lymphoid or mixed-lineage leukemia translocated to, 4
216036_x_at	AK001734	WDTC1	111.48	8.41	49.13	4.38	8.69E-06	WD and tetratricopeptide repeats 1
216242_x_at	AW402635	POLR2J2	291.47	15.60	169.93	9.14	2.79E-05	DNA directed RNA polymerase II polypeptide J-related gene
216442_x_at	AK026737	FN1	10.29	2.47	127.58	40.54	4.43E-05	fibronectin 1
216479_at	AL356414	RPL21	26.99	1.11	21.70	0.95	1.56E-04	ribosomal protein L21
216512_s_at	AL139318	DCT	190.44	52.03	760.26	83.19	7.59E-06	dopachrome delta-isomerase, tyrosine-related protein 2
216513_at	AL139318	DCT	53.51	13.72	309.49	41.51	7.59E-06	dopachrome delta-isomerase, tyrosine-related protein 2
216874_at	U80770		8.33	1.08	74.43	11.38	9.97E-05	Homo sapiens, clone IMAGE:5538654, mRNA
216877_at	U80770		19.01	3.37	134.21	20.09	7.59E-06	Homo sapiens, clone IMAGE:5538654, mRNA
217230_at	AF199015	VIL2	630.45	41.62	327.22	41.02	1.13E-04	villin 2 (ezrin)
217409_at	Z22957	MYO5A	26.87	4.03	122.54	17.80	1.29E-04	myosin VA (heavy polypeptide 12, myoxin)
217432_s_at	AF179281	IDS	129.43	12.66	54.09	9.91	1.41E-04	iduronate 2-sulfatase (Hunter syndrome)
217503_at	AA203487	STK17B	37.24	5.18	14.58	2.38	2.75E-04	serine/threonine kinase 17b
217738_at	BF575514	PBEF1	41.75	8.88	175.46	18.24	7.90E-06	pre-B-cell colony enhancing factor 1
217739_s_at	NM_005746	PBEF1	95.25	12.50	337.55	33.17	8.07E-05	pre-B-cell colony enhancing factor 1
217991_x_at	NM_018070	SSBP3	119.40	10.52	22.41	2.14	3.29E-05	single stranded DNA binding protein 3
217996_at	AA576961	PHLDA1	71.43	11.66	414.92	57.87	9.57E-07	pleckstrin homology-like domain, family A, member 1
217998_at	NM_007350	PHLDA1	195.83	24.96	581.80	61.74	1.41E-04	pleckstrin homology-like domain, family A, member 1
217999_s_at	NM_007350		17.40	2.22	57.34	8.11	9.58E-05	CDNA clone IMAGE:5531727
218010_x_at	NM_024299	C20orf149	141.93	14.42	58.30	4.88	2.11E-05	chromosome 20 open reading frame 149
218095_s_at	NM_018475	TPARL	32.55	2.45	83.11	8.73	1.69E-04	TPA regulated locus
218102_at	NM_015954	DERA	162.60	12.73	346.79	38.57	2.85E-05	2-deoxyribose-5-phosphate aldolase homolog (C. elegans)
218143_s_at	NM_005697	SCAMP2	1295.02	86.34	356.49	41.64	1.60E-04	secretory carrier membrane protein 2
218192_at	NM_016291	IHPK2	368.35	16.09	195.04	18.07	2.15E-04	inositol hexaphosphate kinase 2
218196_at	NM_014028	OSTM1	89.38	13.70	376.20	45.78	1.92E-05	osteopetrosis associated transmembrane protein 1
218205_s_at	NM_017572	MKNK2	2670.86	142.33	1559.75	133.21	2.70E-04	MAP kinase interacting serine/threonine kinase 2
218208_at	NM_025078	PQLC1	1103.67	59.96	732.21	72.65	1.24E-04	PQ loop repeat containing 1
218211_s_at	NM_024101	MLPH	57.60	12.00	631.58	83.27	4.71E-07	melanophilin
218236_s_at	NM_005813	PRKD3	24.39	4.25	132.78	18.24	1.80E-04	protein kinase D3
218350_s_at	NM_015895	GMNN	46.99	6.35	132.49	20.75	1.67E-04	geminin, DNA replication inhibitor
218398_at	NM_016640	MRPS30	56.50	3.64	93.16	6.79	4.51E-05	mitochondrial ribosomal protein S30
218400_at	NM_006187	OAS3	17.63	4.85	50.55	7.78	8.36E-05	2'-5'-oligoadenylate synthetase 3, 100kDa
218402_s_at	NM_022081	HPS4	61.10	5.30	89.29	8.09	2.86E-05	Hermansky-Pudlak syndrome 4
218417_s_at	NM_017842	FLJ20489	65.13	6.87	11.13	1.18	5.80E-05	hypothetical protein FLJ20489
218419_s_at	NM_024107	MGC3123	280.66	24.83	123.22	10.34	4.81E-05	hypothetical protein MGC3123
218456_at	NM_023925	C1QDC1	27.90	4.24	30.32	3.70	1.68E-04	C1q domain containing 1

Probeset	Genbank	Gene Symbol	Nevi-Mean	Nevi-StdErr	Melanoma-Mean	Melanoma-StdErr	p-value	Description
218781_at	NM_024624	SMC6L1	70.31	7.22	188.99	19.05	3.06E-05	SMC6 structural maintenance of chromosomes 6-like 1 (yeast)
218839_at	NM_012258	HEY1	52.98	11.18	242.16	37.53	1.52E-04	hairy/enhancer-of-split related with YRPW motif 1
218880_at	N36408	FOSL2	366.06	42.72	129.07	21.21	1.43E-04	FOS-like antigen 2
218988_at	NM_018656	SLC35E3	15.23	1.66	27.46	3.50	5.73E-05	solute carrier family 35, member E3
219006_at	NM_014165	C6orf66	36.06	4.71	53.26	5.21	9.97E-05	chromosome 6 open reading frame 66
219017_at	NM_018638	ETNK1	24.60	5.42	37.84	3.70	8.80E-05	ethanolamine kinase 1
219098_at	NM_014520	MYBBP1A	87.80	3.81	64.92	3.78	8.69E-06	MYB binding protein (P160) 1a
219142_at	NM_023940	RASL11B	181.76	19.80	31.20	6.52	1.28E-04	RAS-like, family 11, member B
219286_s_at	NM_022768	RBM15	83.96	12.98	158.47	17.00	1.57E-04	RNA binding motif protein 15
219340_s_at	AF123759	CLN8	533.35	53.07	299.63	46.93	2.37E-04	ceroid-lipofuscinosis, neuronal 8
219372_at	NM_014055	CDV1	27.26	4.00	97.00	13.95	1.02E-05	carnitine deficiency-associated, expressed in ventricle 1
219387_at	NM_017571	KIAA1212	74.63	17.14	265.88	28.57	1.58E-05	KIAA1212
219412_at	NM_022337	RAB38	48.02	5.03	151.84	15.99	1.14E-04	RAB38, member RAS oncogene family
219563_at	NM_024633	C14orf139	30.10	4.93	71.93	9.39	1.61E-04	chromosome 14 open reading frame 139
219636_s_at	NM_025139	ARMC9	21.15	3.98	107.45	18.37	8.85E-06	armadillo repeat containing 9
219918_s_at	NM_018123	ASPM	10.85	2.15	28.42	4.74	2.58E-04	asp (abnormal spindle)-like, microcephaly associated
219946_x_at	NM_024729	MYH14	179.53	16.48	48.83	5.30	2.47E-04	myosin, heavy polypeptide 14
220178_at	NM_021731	C19orf28	74.56	19.63	271.67	42.54	3.06E-05	chromosome 19 open reading frame 28
220245_at	NM_016180	SLC45A2	10.68	2.33	102.51	20.97	1.69E-07	solute carrier family 45, member 2
220578_at	NM_025008	TSRC1	41.54	4.98	12.41	1.31	5.26E-05	thrombospondin repeat containing 1
221031_s_at	NM_030817	DKFZP434F0318	10.33	1.14	92.93	30.52	1.37E-05	hypothetical protein DKFZp434F0318
221485_at	AL035683	B4GALT5	24.90	4.05	106.04	14.97	3.96E-06	UDP-Gal:betaGlcNAc beta 1,4- galactosyltransferase, polypeptide 5
221523_s_at	AL138717	RRAGD	14.71	2.47	71.89	9.49	2.15E-04	Ras-related GTP binding D
221524_s_at	AF272036	RRAGD	26.17	5.14	110.07	13.24	1.90E-04	Ras-related GTP binding D
221652_s_at	AF274950	C12orf11	36.16	3.98	87.74	9.50	5.21E-05	chromosome 12 open reading frame 11
221667_s_at	AF133207	HSPB8	504.24	48.75	203.14	41.54	1.52E-04	heat shock 22kDa protein 8
221823_at	AL565741	LOC90355	16.33	1.61	68.86	11.29	1.64E-04	hypothetical gene supported by AF038182; BC009203
221868_at	AB032981	KIAA1155	27.92	2.37	9.96	0.89	1.90E-04	KIAA1155 protein
221951_at	AI739035	TMEM80	80.82	5.05	44.26	5.65	1.77E-05	transmembrane protein 80
222024_s_at	AK022014	AKAP13	228.18	16.28	101.45	11.30	1.71E-04	A kinase (PRKA) anchor protein 13
222146_s_at	AK026674	TCF4	127.46	13.33	33.59	7.28	1.80E-05	transcription factor 4
222153_at	AK023133	MYEF2	28.94	8.85	169.05	27.05	1.33E-05	myelin expression factor 2
222244_s_at	AK000749	TUG1	350.75	17.84	211.99	27.46	1.64E-04	taurine upregulated gene 1
222294_s_at	AW971415	EIF2C2	112.60	14.29	572.72	68.21	1.05E-06	Eukaryotic translation initiation factor 2C, 2
222297_x_at	AV738806	RPL18	52.29	1.66	40.59	1.55	9.57E-06	Ribosomal protein L18
222405_at	AL573951	HSPC121	22.34	1.60	47.21	5.16	2.25E-04	butyrate-induced transcript 1
222428_s_at	D84223	LARS	34.87	2.92	77.66	9.11	2.73E-04	leucyl-tRNA synthetase
222457_s_at	BC001247	EPLIN	479.98	39.42	1485.57	149.74	2.74E-05	epithelial protein lost in neoplasm beta
222486_s_at	AF060152	ADAMTS1	48.25	10.29	105.96	17.87	1.30E-04	ADAM metallopeptidase with thrombospondin type 1 motif, 1
222608_s_at	AK023208	ANLN	41.19	6.86	95.30	13.51	7.38E-05	anillin, actin binding protein (scraps homolog, Drosophila)
222670_s_at	AW135013	MAFB	515.62	38.29	174.75	24.23	2.46E-05	v-maf musculoaponeurotic fibrosarcoma oncogene homolog B
222771_s_at	BF224052	MYEF2	74.48	19.55	385.78	41.89	1.12E-06	myelin expression factor 2
223065_s_at	BC003074	STARD3NL	15.04	1.86	44.28	6.07	1.07E-04	STARD3 N-terminal like
223072_s_at	U79457	WBP1	50.23	4.00	21.20	2.04	8.16E-05	WW domain binding protein 1
223094_s_at	AF274753	ANKH	57.26	7.28	24.00	3.50	2.63E-04	ankylosis, progressive homolog (mouse)
223101_s_at	BC000018	ARPC5L	71.59	5.06	42.30	6.44	1.27E-04	actin related protein 2/3 complex, subunit 5-like
223179_at	BC005009	YPEL3	200.72	20.63	87.08	17.22	7.67E-05	yippee-like 3 (Drosophila)
223181_at	BC000892	C18orf55	65.50	4.40	107.79	9.71	2.73E-04	chromosome 18 open reading frame 55
223253_at	BC000686	EPDR1	6.60	0.60	42.74	17.22	1.58E-05	ependymin related protein 1 (zebrafish)
223287_s_at	AF146696	FOXP1	224.80	19.66	38.59	7.48	4.54E-05	forkhead box P1
223461_at	AF151073	TBC1D7	196.84	26.53	322.54	43.75	1.56E-04	TBC1 domain family, member 7

Probeset	Genbank	Gene Symbol	Nevi-Mean	Nevi-StdErr	Melanoma-Mean	Melanoma-StdErr	p-value	Description
223601_at	AF131839	OLFM2	103.96	8.11	28.82	3.27	6.64E-05	olfactomedin 2
224511_s_at	BC006405	TXNL5	155.38	12.56	224.97	22.34	2.35E-04	thioredoxin-like 5 ; thioredoxin-like 5
224575_at	BE868361	C3orf10	413.52	17.02	262.00	19.17	2.08E-04	chromosome 3 open reading frame 10
224576_at	AK000752	KIAA1181	84.81	16.07	91.70	13.63	7.28E-05	endoplasmic reticulum-golgi intermediate compartment 32 kDa protein
224609_at	AI264216	SLC44A2	140.46	13.66	47.17	7.93	1.15E-05	solute carrier family 44, member 2
224654_at	BG164358	DDX21	52.18	2.49	94.82	7.45	3.00E-05	DEAD (Asp-Glu-Ala-Asp) box polypeptide 21
224691_at	BE622897	UHMK1	190.13	11.29	363.41	37.72	1.97E-04	U2AF homology motif (UHM) kinase 1
224776_at	BF513102	AGPAT6	315.46	19.60	781.97	76.89	4.05E-05	1-acylglycerol-3-phosphate O-acyltransferase 6
224833_at	BE218980	ETS1	69.09	12.14	356.37	31.79	1.54E-07	v-ets erythroblastosis virus E26 oncogene homolog 1 (avian)
224837_at	AW080845	FOXP1	289.75	19.05	74.55	9.89	8.69E-06	forkhead box P1
224838_at	AK026898	FOXP1	615.90	38.34	158.89	22.99	3.00E-05	forkhead box P1
224991_at	AI819630	CMIP	66.89	7.30	26.92	4.93	1.69E-07	c-Maf-inducing protein
225009_at	AA191708	CKLFSF4	61.28	6.94	19.62	1.52	1.08E-05	chemokine-like factor superfamily 4
225147_at	AL521959	PSCD3	326.07	34.89	1380.83	116.67	3.73E-07	pleckstrin homology, Sec7 and coiled-coil domains 3
225175_s_at	AI569503	SLC44A2	692.17	72.17	203.92	35.34	2.08E-04	solute carrier family 44, member 2
225202_at	BE620739	RHOBTB3	44.06	6.64	233.03	39.19	2.79E-04	Rho-related BTB domain containing 3
225395_s_at	AI309997	C9orf10OS	96.54	8.54	37.05	3.23	2.59E-04	chromosome 9 open reading frame 10 opposite strand
225415_at	AA577672	DTX3L	11.25	1.01	23.11	2.50	2.25E-04	deltex 3-like (Drosophila)
225425_s_at	AV726260	MRPL41	175.25	9.36	103.16	6.73	8.41E-05	mitochondrial ribosomal protein L41
225532_at	AI889160	CABLES1	133.90	13.68	422.07	48.88	2.54E-05	Cdk5 and Abl enzyme substrate 1
225581_s_at	BG028213	MRPL50	122.11	9.64	106.41	14.16	1.80E-04	mitochondrial ribosomal protein L50
225600_at	AW303300		28.93	8.29	38.58	6.73	1.50E-05	MRNA; cDNA DKFZp779L1068 (from clone DKFZp779L1068)
225622_at	AI860212	PAG1	60.10	6.57	167.83	15.08	7.38E-05	phosphoprotein associated with glycosphingolipid microdomains 1
225626_at	AK000680	PAG1	143.20	18.12	430.53	39.43	2.48E-04	phosphoprotein associated with glycosphingolipid microdomains 1
225673_at	BE908995	MYADM	98.93	9.75	236.71	32.65	1.23E-04	myeloid-associated differentiation marker
225685_at	AI801777	CDC42EP3	59.02	8.69	218.79	23.44	1.19E-04	CDC42 effector protein (Rho GTPase binding) 3
225817_at	AB051536	CGNL1	136.21	12.65	21.74	4.90	4.94E-05	cingulin-like 1
225842_at	AK026181		104.48	17.75	626.22	73.77	3.73E-07	CDNA clone IMAGE:5531727
225946_at	BG484552	RASSF8	11.13	1.39	63.20	9.78	1.59E-04	Ras association (RalGDS/AF-6) domain family 8
226034_at	BE222344		78.29	22.57	475.04	64.38	4.25E-05	Homo sapiens, clone IMAGE:3881549, mRNA
226054_at	AA702437	BRD4	2300.14	74.52	1285.00	79.52	6.58E-05	bromodomain containing 4
226206_at	BG231691	MAFK	197.62	12.91	78.79	9.16	1.71E-04	v-maf musculoaponeurotic fibrosarcoma oncogene homolog K
226262_at	AA534526	DHX33	55.24	8.31	95.33	10.86	1.01E-04	DEAH (Asp-Glu-Ala-His) box polypeptide 33
226301_at	AV729072	C6orf192	19.27	1.83	88.70	11.38	2.11E-05	chromosome 6 open reading frame 192
226419_s_at	AA046439	SFRS1	133.25	15.63	302.36	35.06	2.10E-04	Splicing factor, arginine/serine-rich 1
226423_at	AW006774	PAQR8	11.58	1.37	26.83	2.90	7.59E-06	progestin and adipoQ receptor family member VIII
226463_at	AW241758	ATP6V1C1	16.92	1.25	42.78	4.87	8.78E-05	ATPase, H+ transporting, lysosomal 42kDa, V1 subunit C, isoform 1
226641_at	AU157224	LOC91526	50.64	6.72	277.30	40.55	1.37E-05	Ankyrin repeat domain 44
226711_at	BF590117	HTLF	48.34	5.36	194.50	23.84	1.37E-05	human T-cell leukemia virus enhancer factor
226893_at	AW173164	ABL2	152.56	21.08	537.96	80.75	3.41E-05	V-abl Abelson murine leukemia viral oncogene homolog 2
226899_at	AK022859	UNC5B	390.64	46.03	45.28	8.59	2.21E-04	unc-5 homolog B (C. elegans)
226965_at	BF438017	FLJ34969	95.48	6.79	178.92	17.28	2.18E-04	hypothetical protein FLJ34969
226988_s_at	AI709055	MYH14	213.54	20.26	49.93	7.61	5.38E-05	myosin, heavy polypeptide 14
227080_at	AW003092	ZNF697	74.53	12.92	227.78	38.44	1.65E-05	zinc finger protein 697
227098_at	BC004110	DUSP18	62.83	6.01	17.13	1.50	2.36E-04	dual specificity phosphatase 18
227099_s_at	AW276078	LOC387763	68.25	8.37	540.61	80.01	4.39E-06	hypothetical LOC387763
227239_at	AV734839	DRCTNNB1A	14.87	1.94	84.20	12.07	3.50E-05	down-regulated by Ctnnb1, a
227354_at	BF589359	PAG1	12.13	1.38	39.47	5.02	1.90E-04	phosphoprotein associated with glycosphingolipid microdomains 1
227396_at	AI631833		67.81	14.47	290.83	30.20	1.60E-04	Homo sapiens, clone IMAGE:4454331, mRNA
227698_s_at	AW007215	RAB40C	276.42	37.98	95.36	15.03	2.56E-04	RAB40C, member RAS oncogene family
227761_at	AW235548	MYO5A	161.67	18.59	557.29	46.43	2.51E-06	myosin VA (heavy polypeptide 12, myoxin)

Probeset	Genbank	Gene Symbol	Nevi-Mean	Nevi-StdErr	Melanoma-Mean	Melanoma-StdErr	p-value	Description
227870_at	AB046848	NOPE	11.97	3.66	97.66	18.94	1.80E-07	likely ortholog of mouse neighbor of Punc E11
227950_at	AI829920	UBE2H	159.03	19.53	67.95	14.72	2.35E-04	Ubiquitin-conjugating enzyme E2H (UBC8 homolog, yeast)
227994_x_at	AA548838	C20orf149	469.01	42.41	226.11	18.39	4.99E-06	chromosome 20 open reading frame 149
228016_s_at	AI858055		57.14	4.64	33.12	5.08	1.04E-04	Hypothetical LOC400843
228095_at	AA608749	PHF14	84.34	7.27	148.91	17.58	2.10E-04	PHD finger protein 14
228135_at	AA738437	C1orf52	195.56	9.92	295.34	22.42	2.15E-04	chromosome 1 open reading frame 52
228156_at	AW342078		53.52	4.05	33.38	6.11	5.60E-05	Homo sapiens, clone IMAGE:4346533, mRNA
228245_s_at	AW594320	OVOS2	150.97	31.80	1313.58	132.36	1.39E-07	ovostatin 2
228415_at	AA205444	AP1S2	43.10	3.40	151.11	31.93	3.56E-05	Adaptor-related protein complex 1, sigma 2 subunit
228428_at	AA521285	EEIG1	107.66	7.48	56.57	5.29	5.09E-05	Chromosome 9 open reading frame 132
228496_s_at	AW243081	CRIM1	874.60	67.63	166.61	37.66	1.60E-04	Cysteine rich transmembrane BMP regulator 1 (chordin-like)
228519_x_at	AW027567	CIRBP	380.40	21.40	199.75	6.95	9.97E-05	cold inducible RNA binding protein
228768_at	N51056	KIAA1961	99.91	6.71	206.72	19.39	9.29E-05	KIAA1961 gene
228919_at	AA601031	CDC2L2	748.95	65.62	137.24	36.01	1.39E-06	cell division cycle 2-like 1 (PITSLRE proteins)
228942_s_at	BE858624	DAB2IP	261.72	33.80	75.93	13.22	2.11E-05	DAB2 interacting protein
229017_s_at	N31717	RIPK5	351.33	39.75	1013.37	108.60	1.08E-04	receptor interacting protein kinase 5
229143_at	AW449353	CNOT3	41.67	4.41	24.39	3.47	2.59E-04	CCR4-NOT transcription complex, subunit 3
229574_at	AI268231	TRA2A	372.34	23.30	180.71	16.90	8.48E-06	Transformer-2 alpha
229713_at	AW665227		34.86	4.38	132.91	17.72	4.82E-05	CDNA FLJ13267 fis, clone OVARC1000964
229797_at	AI636080	MCOLN3	72.67	22.01	413.99	61.13	1.33E-04	mucolipin 3
229800_at	AI129626	DCAMKL1	28.90	7.65	186.67	37.51	2.70E-04	Doublecortin and CaM kinase-like 1
230207_s_at	AI692645	DOCK5	25.94	3.60	90.34	14.70	4.31E-05	Dedicator of cytokinesis 5
230263_s_at	BF447954	DOCK5	23.83	3.31	94.66	15.17	9.97E-05	dedicator of cytokinesis 5
230264_s_at	AA551090	AP1S2	51.03	9.27	273.57	39.49	1.36E-05	adaptor-related protein complex 1, sigma 2 subunit
230333_at	BE326919	SAT	89.72	24.78	441.83	54.96	1.67E-06	Spermidine/spermine N1-acetyltransferase
230413_s_at	AA480858	AP1S2	23.90	4.30	128.14	17.80	1.01E-04	Adaptor-related protein complex 1, sigma 2 subunit
230513_at	AW104426	BAT3	44.59	5.55	19.82	1.71	7.87E-05	HLA-B associated transcript 3
230741_at	AI655467		62.21	7.76	325.64	64.57	3.17E-05	CDNA FLJ41454 fis, clone BRSTN2011597
230748_at	AI873273	SLC16A6	94.03	15.53	393.71	51.36	1.63E-04	solute carrier family 16, member 6
230801_at	AW300917	C20orf77	33.82	2.69	24.41	1.06	1.68E-04	Chromosome 20 open reading frame 77
231643_s_at	BE045541	CMIP	224.52	18.00	106.78	16.00	7.28E-05	C-Maf-inducing protein
231666_at	AA194168	PAX3	18.22	4.09	109.75	14.16	2.79E-05	paired box gene 3 (Waardenburg syndrome 1)
231778_at	AI769274	DLX3	231.62	39.07	37.50	8.66	2.73E-04	distal-less homeo box 3
231944_at	AL045717	ERO1LB	30.68	4.73	61.97	6.43	1.11E-04	ERO1-like beta (S. cerevisiae)
232382_s_at	BE150929	PCMTD1	601.17	26.60	400.30	24.46	3.50E-05	protein-L-isoaspartate (D-aspartate) O-methyltransferase domain containing 1
232425_at	AK026814	SNX25	66.37	10.68	148.38	23.73	2.87E-04	sorting nexin 25
232504_at	AL389942	LOC285628	27.00	6.53	279.30	35.89	1.12E-06	hypothetical protein LOC285628
232676_x_at	AK002075	MYEF2	86.79	23.46	511.49	51.04	1.69E-07	myelin expression factor 2
232926_x_at	AL041075	ANKRD19	863.90	37.67	602.67	31.71	8.47E-05	ankyrin repeat domain 19
232977_x_at	BC004396	MYH14	228.50	24.26	47.17	6.82	1.10E-04	myosin, heavy polypeptide 14
233540_s_at	AK025867	CDK5RAP2	85.34	9.34	141.89	14.90	1.74E-04	CDK5 regulatory subunit associated protein 2
233571_x_at	AL121829	C20orf149	438.81	34.20	250.32	19.63	2.11E-05	chromosome 20 open reading frame 149
233882_s_at	AK022831	SEMA6D	33.50	8.39	215.75	43.10	3.05E-07	sema domain, 6D
234290_x_at	BC000676	MYH14	242.41	39.15	42.57	6.77	6.64E-05	myosin, heavy polypeptide 14
234339_s_at	AF296124	GLTSCR2	228.87	19.05	75.92	6.46	2.73E-04	glioma tumor suppressor candidate region gene 2
234607_at	AK025002	ARRDC1	80.06	4.00	58.45	3.03	2.29E-04	Arrestin domain containing 1
235020_at	AI366784	TAF4B	17.10	2.30	42.74	5.13	2.08E-05	TAF4b RNA polymerase II, TBP-associated factor
235130_at	AV703394	PANK2	23.66	1.34	34.59	2.53	2.61E-04	Pantothenate kinase 2 (Hallervorden-Spatz syndrome)
235605_at	AI283046		182.52	10.49	71.79	5.72	1.72E-05	Similar to cDNA sequence BC035954
235709_at	H37811	GAS2L3	8.73	1.04	33.26	5.39	1.90E-04	growth arrest-specific 2 like 3
235766_x_at	AA743462	EIF2C2	56.08	7.54	245.79	29.81	1.35E-04	Eukaryotic translation initiation factor 2C, 2

Probeset	Genbank	Gene Symbol	Nevi-Mean	Nevi-StdErr	Melanoma-Mean	Melanoma-StdErr	p-value	Description
236972_at	AI351421	TRIM63	25.06	6.13	333.43	43.79	6.75E-10	tripartite motif-containing 63
237069_s_at	AI277662	TRPM1	32.79	11.55	226.92	27.00	3.73E-07	transient receptor potential cation channel, subfamily M, member 1
237070_at	AI277662	TRPM1	54.37	11.39	688.96	87.21	8.58E-09	transient receptor potential cation channel, subfamily M, member 1
237464_at	AI241501	IMAA	22.08	3.47	52.34	6.97	5.52E-06	LAT1-3TM protein 2
237737_at	AI359676	LOC375010	13.82	1.46	73.33	9.36	2.90E-05	hypothetical LOC375010 ; hypothetical LOC401131
238756_at	AI860012	GAS2L3	15.69	3.41	105.33	14.87	2.53E-05	Growth arrest-specific 2 like 3
239197_s_at	BG470312	EZH1	142.21	9.53	68.17	9.30	1.11E-04	enhancer of zeste homolog 1 (Drosophila)
239606_at	AA669135	GCNT2	16.42	2.10	91.05	12.75	2.11E-05	Glucosaminyl (N-acetyl) transferase 2, I-branching enzyme
241129_at	AA059398	ALS2CR2	20.77	4.38	67.25	10.80	1.62E-04	Amyotrophic lateral sclerosis 2 chromosome region, candidate 2
241180_at	AV660057	THRAP1	41.31	5.83	52.80	6.25	2.73E-04	Thyroid hormone receptor associated protein 1
241435_at	AA702930	ETS1	25.98	4.19	125.37	17.35	2.10E-05	V-ets erythroblastosis virus E26 oncogene homolog 1 (avian)
241898_at	AA991267		126.58	10.41	42.18	4.54	2.30E-07	similar to KIAA0825 protein
241957_x_at	AI686521	LIN7B	39.76	3.19	14.43	1.66	1.13E-04	lin-7 homolog B (C. elegans)
242038_at	BG037106	LRRC8B	291.69	32.48	86.83	13.46	1.90E-04	leucine rich repeat containing 8 family, member B
242049_s_at	BE783098	NAG	186.09	12.27	109.74	14.76	2.14E-05	neuroblastoma-amplified protein
242321_at	AI628689		85.37	8.05	28.47	4.69	1.15E-04	CDNA FLJ32401 fis, clone SKMUS2000339
243720_at	AA039576	CMIP	193.37	17.58	88.88	11.84	2.46E-05	C-Maf-inducing protein
244235_at	AW273860	IVNS1ABP	23.72	1.56	73.83	7.69	1.79E-04	influenza virus NS1A binding protein
244358_at	AW372457		61.75	7.48	15.43	2.28	7.22E-05	cdna:Genscan chromosome:GRCh37:6:108943859:108944134:1
244829_at	N44676	MGC40222	23.68	9.23	376.13	45.89	4.60E-11	Hypothetical protein MGC40222
244845_at	BF725383		220.62	19.06	35.67	4.82	1.52E-04	CDNA FLJ45435 fis, clone BRHIP3042817
32259_at	AB002386	EZH1	73.09	6.39	32.57	3.26	7.09E-05	enhancer of zeste homolog 1 (Drosophila)
44783_s_at	R61374	HEY1	222.06	36.40	1154.01	152.39	1.49E-04	hairy/enhancer-of-split related with YRPW motif 1
45653_at	AW026481	KCTD13	32.32	1.83	24.55	1.81	2.38E-04	potassium channel tetramerisation domain containing 13
54037_at	AL041451	HPS4	104.45	6.15	213.68	18.08	6.30E-05	Hermansky-Pudlak syndrome 4

Table S3. Table of 168 genes identified by the TreeNet analysis in the training set

Probeset	Genbank	Gene Symbol	Nevi-Mean	Nevi-StdErr	Melanoma-Mean	Melanoma-StdErr	p-value	Description
1555167_s_at	BC020691	PBEF1	143.35	27.54	475.57	66.14	2.11E-05	pre-B-cell colony enhancing factor 1
1555505_a_at	BC027179	TYR	59.07	18.39	359.59	53.14	7.13E-06	tyrosinase (oculocutaneous albinism IA)
1555579_s_at	BC029442	PTPRM	65.71	17.99	430.62	47.44	2.36E-07	protein tyrosine phosphatase, receptor type, M
1556988_s_at	BE220618	CHD1L	45.24	4.32	82.79	8.32	8.59E-05	chromodomain helicase DNA binding protein 1-like
1557292_a_at	AW665790	MCOLN3	53.73	13.95	203.04	26.96	3.86E-05	mucolipin 3
1557797_a_at	AW611486	ZFHX1B	119.29	32.81	736.29	83.12	3.73E-07	Zinc finger homeobox 1b
1559397_s_at	BE788667	MGC3121	222.18	27.01	64.50	14.35	4.04E-07	hypothetical protein MGC3121
1559759_at	BE407830	KIFC3	33.77	3.75	16.91	1.41	5.30E-05	Kinesin family member C3
200601_at	U48734	ACTN4	507.22	37.69	126.67	14.80	3.05E-07	actinin, alpha 4
200645_at	NM_007278	GABARAP	1207.88	52.96	609.53	68.45	3.50E-05	GABA(A) receptor-associated protein
200665_s_at	NM_003118	SPARC	161.23	24.84	840.08	107.62	1.31E-07	secreted protein, acidic, cysteine-rich (osteonectin)
200747_s_at	NM_006185	NUMA1	117.66	6.55	61.99	4.79	1.67E-04	nuclear mitotic apparatus protein 1
200755_s_at	BF939365	CALU	215.37	18.31	480.09	61.73	9.97E-05	calumenin
200782_at	NM_001154	ANXA5	168.01	24.87	602.76	64.31	3.14E-05	annexin A5
200819_s_at	NM_001018	RPS15	3484.70	171.23	1321.29	108.41	4.04E-07	ribosomal protein S15
200914_x_at	BF589024	KTN1	401.66	22.08	258.91	24.14	5.80E-05	kinectin 1 (kinesin receptor)
200958_s_at	NM_005625	SDCBP	531.24	85.85	2685.15	190.75	3.11E-07	syndecan binding protein (syntenin)
201245_s_at	AL523776	OTUB1	253.01	40.00	65.66	8.60	9.45E-05	OTU domain, ubiquitin aldehyde binding 1
201299_s_at	NM_018221	MOBK1B	663.01	27.96	397.14	33.55	2.04E-04	MOB1, Mps One Binder kinase activator-like 1B (yeast)
201423_s_at	AL037208	CUL4A	67.56	4.63	92.51	8.24	1.74E-04	cullin 4A
201470_at	NM_004832	GSTO1	330.19	26.44	820.41	79.07	1.98E-04	glutathione S-transferase omega 1
201556_s_at	BC002737	VAMP2	816.64	46.55	374.33	37.79	2.46E-06	vesicle-associated membrane protein 2 (synaptobrevin 2)
201565_s_at	NM_002166	ID2	302.26	41.96	63.72	9.93	6.36E-05	inhibitor of DNA binding 2, dominant negative helix-loop-helix protein
201603_at	AI817061	PPP1R12A	243.40	16.62	388.04	25.47	3.85E-05	protein phosphatase 1, regulatory (inhibitor) subunit 12A
201605_x_at	NM_004368	CNN2	335.60	19.03	173.58	10.47	1.51E-05	calponin 2
201739_at	NM_005627	SGK	2806.10	150.55	5196.49	323.88	1.23E-04	serum/glucocorticoid regulated kinase
201745_at	NM_002822	PTK9	93.82	7.82	202.86	20.72	3.29E-05	PTK9 protein tyrosine kinase 9
201945_at	NM_002569	FURIN	534.63	53.88	197.37	32.99	4.31E-05	furin (paired basic amino acid cleaving enzyme)
201976_s_at	NM_012334	MYO10	548.20	30.76	1083.45	83.87	1.54E-04	myosin X
202088_at	AI635449	SLC39A6	433.84	35.97	1152.25	84.20	6.31E-05	solute carrier family 39 (zinc transporter), member 6
202158_s_at	NM_006561	CUGBP2	44.46	9.61	176.72	19.61	7.89E-05	CUG triplet repeat, RNA binding protein 2
202370_s_at	NM_001755	CBFB	144.37	13.05	198.16	20.44	9.97E-05	core-binding factor, beta subunit
202450_s_at	NM_000396	CTSK	72.84	9.69	286.30	43.78	4.40E-06	cathepsin K (pycnodysostosis)
202478_at	NM_021643	TRIB2	181.01	49.12	888.22	91.35	2.22E-08	tribbles homolog 2 (Drosophila)
202479_s_at	BC002637	TRIB2	120.79	28.52	531.08	66.94	1.54E-04	tribbles homolog 2 (Drosophila)
202572_s_at	NM_014902	DLGAP4	386.81	26.15	220.17	26.80	4.89E-05	discs, large (Drosophila) homolog-associated protein 4
202589_at	NM_001071	TYMS	45.64	5.55	101.47	15.82	5.65E-05	thymidylate synthetase
202984_s_at	AA457021	BAG5	101.97	6.07	49.21	4.78	2.90E-05	BCL2-associated athanogene 5
203004_s_at	NM_005920	MEF2D	24.90	3.15	12.75	2.12	2.69E-04	MADS box transcription enhancer factor 2, polypeptide D
203217_s_at	NM_003896	ST3GAL5	47.73	11.12	253.50	31.94	1.78E-05	ST3 beta-galactoside alpha-2,3-sialyltransferase 5
203455_s_at	NM_002970	SAT	867.30	117.46	3602.58	239.90	3.59E-05	spermidine/spermine N1-acetyltransferase
203603_s_at	NM_014795	ZFHX1B	61.21	15.90	311.12	35.03	8.53E-06	zinc finger homeobox 1b

Probeset ID: Probeset ID from Affymetrix U133 plus 2.0 array
GeneBank: GeneBank accession number
Nevi-mean: average expression value of nevi samples
Nevi-StdErr: standard error of nevi samples
Melanoma-mean: average expression value of melanoma samples
Melanoma-StdErr: standard error of melanoma samples
p-value: t-test ($p < 0.05$, Westfall and Young permutation multi-testing correction, false discovery rate, $q<0.05$)

Probeset	Genbank	Gene Symbol	Nevi-Mean	Nevi-StdErr	Melanoma-Mean	Melanoma-StdErr	p-value	Description
203735_x_at	N35896	PPFIBP1	1051.06	53.76	720.76	25.77	8.66E-06	PTPRF interacting protein, binding protein 1 (liprin beta 1)
204014_at	NM_001394	DUSP4	104.15	35.60	604.41	111.04	9.18E-05	dual specificity phosphatase 4
204086_at	NM_006115	PRAME	7.54	1.80	40.93	6.11	1.44E-06	preferentially expressed antigen in melanoma
204271_s_at	M74921	EDNRB	385.40	106.32	2757.37	286.01	1.25E-09	endothelin receptor type B
204470_at	NM_001511	CXCL1	29.15	15.59	326.34	60.65	3.33E-06	chemokine (C-X-C motif) ligand 1
204527_at	NM_000259	MYO5A	43.13	6.08	141.26	15.81	4.04E-07	myosin VA (heavy polypeptide 12, myoxin)
205051_s_at	NM_000222	KIT	111.95	24.27	926.32	91.49	8.58E-09	v-kit Hardy-Zuckerman 4 feline sarcoma viral oncogene homolog
205260_s_at	NM_001107	ACYP1	87.40	13.65	126.82	16.15	4.51E-05	acylphosphatase 1, erythrocyte (common) type
205681_at	NM_004049	BCL2A1	23.59	6.12	191.78	28.24	1.56E-05	BCL2-related protein A1
205853_at	NM_015872	ZBTB7B	23.55	1.34	15.92	0.81	2.06E-05	zinc finger and BTB domain containing 7B
206180_x_at	NM_023931	MGC2474	374.29	27.72	203.26	10.60	2.17E-05	hypothetical protein MGC2474
206376_at	NM_018057	SLC6A15	15.83	3.47	118.09	16.25	2.51E-06	solute carrier family 6, member 15
206701_x_at	NM_003991	EDNRB	97.32	30.82	622.88	90.02	1.06E-08	endothelin receptor type B
208073_x_at	NM_003316	TTC3	829.04	46.89	2365.96	180.22	3.50E-05	tetratricopeptide repeat domain 3
208738_x_at	AK024823	SUMO2	1255.22	48.09	958.83	63.14	1.26E-05	SMT3 suppressor of mif two 3 homolog 2 (yeast)
209000_s_at	BC001329	SEPT8	400.44	31.30	204.32	28.57	3.45E-05	septin 8
209023_s_at	BC001765	STAG2	225.77	14.59	139.32	11.13	2.19E-04	stromal antigen 2
209058_at	AB002282	EDF1	1145.69	68.22	404.32	38.59	3.50E-05	endothelial differentiation-related factor 1
209168_at	AW148844	GPM6B	50.25	7.43	175.28	18.56	1.69E-07	glycoprotein M6B
209234_at	BF939474	KIF1B	752.31	42.75	394.00	38.98	4.18E-05	kinesin family member 1B
209238_at	BE966922	STX3A	58.81	6.06	90.71	10.26	2.32E-06	syntaxin 3A
209241_x_at	AB041926	MINK1	35.94	2.28	23.30	1.93	9.97E-05	misshapen-like kinase 1 (zebrafish)
209514_s_at	BE502030	RAB27A	90.54	10.86	410.18	50.07	2.00E-05	RAB27A, member RAS oncogene family
209780_at	AL136883	PHTF2	374.25	24.36	743.29	49.95	2.70E-04	putative homeodomain transcription factor 2
209814_at	BC004421	ZNF330	349.02	28.65	703.05	50.98	1.24E-04	zinc finger protein 330
209953_s_at	U63131	CDC37	61.43	5.73	32.46	3.70	5.99E-06	CDC37 cell division cycle 37 homolog
210020_x_at	M58026	CALML3	1841.79	196.75	326.59	47.71	1.90E-04	calmodulin-like 3
210198_s_at	BC002665	PLP1	40.67	11.55	233.17	28.92	3.73E-07	proteolipid protein 1
210592_s_at	M55580	SAT	885.24	140.83	3578.58	281.98	1.21E-05	spermidine/spermine N1-acetyltransferase
210852_s_at	AF229180	AASS	35.01	3.17	91.87	11.78	1.29E-05	aminoadipate-semialdehyde synthase
210951_x_at	AF125393	RAB27A	86.21	10.75	400.09	44.52	4.15E-05	RAB27A, member RAS oncogene family
211084_x_at	Z25429	PRKD3	69.08	12.36	197.63	23.77	4.90E-05	protein kinase D3 ; protein kinase D3
211347_at	AF064105	CDC14B	100.33	9.39	310.91	35.14	1.02E-05	CDC14 cell division cycle 14 homolog B
211388_s_at	U80761	U80761	325.06	23.06	141.78	23.32	4.31E-05	Homo sapiens CTG26 alternate open reading frame mRNA
211546_x_at	L36674	SNCA	78.25	9.01	293.34	30.20	7.11E-06	synuclein, alpha
211905_s_at	AF011375	ITGB4	95.72	15.39	35.32	3.47	2.02E-04	integrin, beta 4
211948_x_at	BG261071	BAT2D1	212.80	13.59	366.41	31.34	4.39E-06	BAT2 domain containing 1
212038_s_at	AL515918	VDAC1	963.19	52.79	1257.05	81.82	1.26E-05	voltage-dependent anion channel 1
212138_at	AK021757	SCC-112	104.48	6.84	165.96	15.20	8.66E-06	SCC-112 protein
212382_at	BF433429	TCF4	575.38	52.61	100.45	18.44	6.39E-05	Transcription factor 4
213119_at	AW058600	SLC36A1	110.04	6.95	168.00	11.54	1.14E-05	solute carrier family 36 (proton/amino acid symporter), member 1
213146_at	AA521267	JMJD3	231.66	13.18	102.86	8.34	4.90E-05	jumonji domain containing 3
213688_at	N25325	CALM1	205.48	14.33	88.78	10.39	1.72E-04	calmodulin 1 (phosphorylase kinase, delta)
214681_at	AI830490	GK	49.40	9.48	171.99	18.10	1.89E-06	glycerol kinase
216036_x_at	AK001734	WDTC1	111.48	8.41	49.13	4.38	8.69E-06	WD and tetratricopeptide repeats 1
216242_x_at	AW402635	POLR2J2	291.47	15.60	169.93	9.14	2.79E-05	DNA directed RNA polymerase II polypeptide J-related gene
216479_at	AL356414	RPL21	26.99	1.11	21.70	0.95	1.56E-04	ribosomal protein L21
216512_s_at	AL139318	DCT	190.44	52.03	760.26	83.19	7.59E-06	dopachrome delta-isomerase, tyrosine-related protein 2
216513_at	AL139318	DCT	53.51	13.72	309.49	41.51	7.59E-06	dopachrome delta-isomerase, tyrosine-related protein 2
217230_at	AF199015	VIL2	630.45	41.62	327.22	41.02	1.13E-04	villin 2 (ezrin)
217503_at	AA203487	STK17B	37.24	5.18	14.58	2.38	2.75E-04	serine/threonine kinase 17b
217738_at	BF575514	PBEF1	41.75	8.88	175.46	18.24	7.90E-06	pre-B-cell colony enhancing factor 1
217996_at	AA576961	PHLDA1	71.43	11.66	414.92	57.87	9.57E-07	pleckstrin homology-like domain, family A, member 1

Probeset	Genbank	Gene Symbol	Nevi-Mean	Nevi-StdErr	Melanoma-Mean	Melanoma-StdErr	p-value	Description
218102_at	NM_015954	DERA	162.60	12.73	346.79	38.57	2.85E-05	2-deoxyribose-5-phosphate aldolase homolog (C. elegans)
218402_s_at	NM_022081	HPS4	61.10	5.30	89.29	8.09	2.86E-05	Hermansky-Pudlak syndrome 4
218419_s_at	NM_024107	MGC3123	280.66	24.83	123.22	10.34	4.81E-05	hypothetical protein MGC3123
219017_at	NM_018638	ETNK1	24.60	5.42	37.84	3.70	8.80E-05	ethanolamine kinase 1
219098_at	NM_014520	MYBBP1A	87.80	3.81	64.92	3.78	8.69E-06	MYB binding protein (P160) 1a
219372_at	NM_014055	CDV1	27.26	4.00	97.00	13.95	1.02E-05	carnitine deficiency-associated, expressed in ventricle 1
219387_at	NM_017571	KIAA1212	74.63	17.14	265.88	28.57	1.58E-05	KIAA1212
219636_s_at	NM_025139	ARMC9	21.15	3.98	107.45	18.37	8.85E-06	armadillo repeat containing 9
219946_x_at	NM_024729	MYH14	179.53	16.48	48.83	5.30	2.47E-04	myosin, heavy polypeptide 14
220245_at	NM_016180	SLC45A2	10.68	2.33	102.51	20.97	1.69E-07	solute carrier family 45, member 2
220578_at	NM_025008	TSRC1	41.54	4.98	12.41	1.31	5.26E-05	thrombospondin repeat containing 1
221031_s_at	NM_030817	DKFZP434F0318	10.33	1.14	92.93	30.52	1.37E-05	hypothetical protein DKFZp434F0318
221667_s_at	AF133207	HSPB8	504.24	48.75	203.14	41.54	1.52E-04	heat shock 22kDa protein 8
221951_at	AI739035	TMEM80	80.82	5.05	44.26	5.65	1.77E-05	transmembrane protein 80
222146_s_at	AK026674	TCF4	127.46	13.33	33.59	7.28	1.80E-05	transcription factor 4
222297_x_at	AV738806	RPL18	52.29	1.66	40.59	1.55	9.57E-06	Ribosomal protein L18
222670_s_at	AW135013	MAFB	515.62	38.29	174.75	24.23	2.46E-05	v-maf musculoaponeurotic fibrosarcoma oncogene homolog B
223065_s_at	BC003074	STARD3NL	15.04	1.86	44.28	6.07	1.07E-04	STARD3 N-terminal like
223101_s_at	BC000018	ARPC5L	71.59	5.06	42.30	6.44	1.27E-04	actin related protein 2/3 complex, subunit 5-like
223253_at	BC000686	EPDR1	6.60	0.60	42.74	17.22	1.58E-05	ependymin related protein 1 (zebrafish)
223461_at	AF151073	TBC1D7	196.84	26.53	322.54	43.75	1.56E-04	TBC1 domain family, member 7
223601_at	AF131839	OLFM2	103.96	8.11	28.82	3.27	6.64E-05	olfactomedin 2
224576_at	AK000752	KIAA1181	84.81	16.07	91.70	13.63	7.28E-05	endoplasmic reticulum-golgi intermediate compartment 32 kDa protein
224609_at	AI264216	SLC44A2	140.46	13.66	47.17	7.93	1.15E-05	solute carrier family 44, member 2
224654_at	BG164358	DDX21	52.18	2.49	94.82	7.45	3.00E-05	DEAD (Asp-Glu-Ala-Asp) box polypeptide 21 ; zinc finger protein 596
224691_at	BE622897	UHMK1	190.13	11.29	363.41	37.72	1.97E-04	U2AF homology motif (UHM) kinase 1
224833_at	BE218980	ETS1	69.09	12.14	356.37	31.79	1.54E-07	v-ets erythroblastosis virus E26 oncogene homolog 1
224991_at	AI819630	CMIP	66.89	7.30	26.92	4.93	1.69E-07	c-Maf-inducing protein
225009_at	AA191708	CKLFSF4	61.28	6.94	19.62	1.52	1.08E-05	chemokine-like factor superfamily 4
225147_at	AL521959	PSCD3	326.07	34.89	1380.83	116.67	3.73E-07	pleckstrin homology, Sec7 and coiled-coil domains 3
225581_s_at	BG028213	MRPL50	122.11	9.64	106.41	14.16	1.80E-04	mitochondrial ribosomal protein L50
225600_at	AW303300	DKFZp779L1068	28.93	8.29	38.58	6.73	1.50E-05	MRNA; cDNA DKFZp779L1068
225842_at	AK026181		104.48	17.75	626.22	73.77	3.73E-07	CDNA clone IMAGE:5531727
226034_at	BE222344		78.29	22.57	475.04	64.38	4.25E-05	Homo sapiens, clone IMAGE:3881549, mRNA
226054_at	AA702437	BRD4	2300.14	74.52	1285.00	79.52	6.58E-05	bromodomain containing 4
226206_at	BG231691	MAFK	197.62	12.91	78.79	9.16	1.71E-04	v-maf musculoaponeurotic fibrosarcoma oncogene homolog K
226262_at	AA534526	DHX33	55.24	8.31	95.33	10.86	1.01E-04	DEAH (Asp-Glu-Ala-His) box polypeptide 33
226301_at	AV729072	C6orf192	19.27	1.83	88.70	11.38	2.11E-05	chromosome 6 open reading frame 192
226893_at	AW173164	ABL2	152.56	21.08	537.96	80.75	3.41E-05	V-abl Abelson murine leukemia viral oncogene homolog 2
226988_s_at	AI709055	MYH14	213.54	20.26	49.93	7.61	5.38E-05	myosin, heavy polypeptide 14
227099_s_at	AW276078	LOC387763	68.25	8.37	540.61	80.01	4.39E-06	hypothetical LOC387763
227396_at	AI631833		67.81	14.47	290.83	30.20	1.60E-04	Homo sapiens, clone IMAGE:4454331, mRNA
227870_at	AB046848	NOPE	11.97	3.66	97.66	18.94	1.80E-07	likely ortholog of mouse neighbor of Punc E11
227994_x_at	AA548838	C20orf149	469.01	42.41	226.11	18.39	4.99E-06	chromosome 20 open reading frame 149
228156_at	AW342078		53.52	4.05	33.38	6.11	5.60E-05	Homo sapiens, clone IMAGE:4346533, mRNA
228415_at	AA205444	AP1S2	43.10	3.40	151.11	31.93	3.56E-05	Adaptor-related protein complex 1, sigma 2 subunit
228428_at	AA521285	EEIG1	107.66	7.48	56.57	5.29	5.09E-05	Chromosome 9 open reading frame 132
228519_x_at	AW027567	CIRBP	380.40	21.40	199.75	6.95	9.97E-05	cold inducible RNA binding protein
228768_at	N51056	KIAA1961	99.91	6.71	206.72	19.39	9.29E-05	KIAA1961 gene
229574_at	AI268231	TRA2A	372.34	23.30	180.71	16.90	8.48E-06	Transformer-2 alpha
229713_at	AW665227		34.86	4.38	132.91	17.72	4.82E-05	CDNA FLJ13267 fis, clone OVARC1000964
230333_at	BE326919	SAT	89.72	24.78	441.83	54.96	1.67E-06	Spermidine/spermine N1-acetyltransferase
231643_s_at	BE045541	CMIP	224.52	18.00	106.78	16.00	7.28E-05	C-Maf-inducing protein

Probeset	Genbank	Gene Symbol	Nevi-Mean	Nevi-StdErr	Melanoma-Mean	Melanoma-StdErr	p-value	Description
231944_at	AL045717	ERO1LB	30.68	4.73	61.97	6.43	1.11E-04	ERO1-like beta (S. cerevisiae)
232926_x_at	AL041075	ANKRD19	863.90	37.67	602.67	31.71	8.47E-05	ankyrin repeat domain 19
233540_s_at	AK025867	CDK5RAP2	85.34	9.34	141.89	14.90	1.74E-04	CDK5 regulatory subunit associated protein 2
233882_s_at	AK022831	SEMA6D	33.50	8.39	215.75	43.10	3.05E-07	sema domain 6D
234607_at	AK025002	ARRDC1	80.06	4.00	58.45	3.03	2.29E-04	Arrestin domain containing 1
236972_at	AI351421	TRIM63	25.06	6.13	333.43	43.79	6.75E-10	tripartite motif-containing 63
237464_at	AI241501	IMAA	22.08	3.47	52.34	6.97	5.52E-06	LAT1-3TM protein 2
237737_at	AI359676	LOC375010	13.82	1.46	73.33	9.36	2.90E-05	hypothetical LOC375010
238756_at	AI860012	GAS2L3	15.69	3.41	105.33	14.87	2.53E-05	Growth arrest-specific 2 like 3
239606_at	AA669135	GCNT2	16.42	2.10	91.05	12.75	2.11E-05	Glucosaminyl (N-acetyl) transferase 2, I-branching enzyme
241180_at	AV660057	THRAP1	41.31	5.83	52.80	6.25	2.73E-04	Thyroid hormone receptor associated protein 1
241435_at	AA702930	ETS1	25.98	4.19	125.37	17.35	2.10E-05	V-ets erythroblastosis virus E26 oncogene homolog 1 (avian)
241898_at	AA991267		126.58	10.41	42.18	4.54	2.30E-07	Transcribed locus, moderately similar to XP_517655.1 PREDICTED
242049_s_at	BE783098	NAG	186.09	12.27	109.74	14.76	2.14E-05	neuroblastoma-amplified protein
242321_at	AI628689		85.37	8.05	28.47	4.69	1.15E-04	CDNA FLJ32401 fis, clone SKMUS2000339
243720_at	AA039576	CMIP	193.37	17.58	88.88	11.84	2.46E-05	C-Maf-inducing protein
244358_at	AW372457		61.75	7.48	15.43	2.28	7.22E-05	cdna:Genscan chromosome:GRCh37:6:108943859:108944134:1
244829_at	N44676	MGC40222	23.68	9.23	376.13	45.89	4.60E-11	Hypothetical protein MGC40222
32259_at	AB002386	EZH1	73.09	6.39	32.57	3.26	7.09E-05	enhancer of zeste homolog 1 (Drosophila)
45653_at	AW026481	KCTD13	32.32	1.83	24.55	1.81	2.38E-04	potassium channel tetramerisation domain containing 13

Table S4. Description of the 17-gene melanoma classifier

Probeset	Genbank	Gene Symbol	Nevi-Mean	Nevi-StdErr	Melanoma-Mean	Melanoma-StdErr	p-value	Description
200601_at	U48734	ACTN4	507.22	37.69	126.67	14.80	3.05E-07	actinin, alpha 4
200819_s_at	NM_001018	RPS15	3484.70	171.23	1321.29	108.41	4.04E-07	ribosomal protein S15
201605_x_at	NM_004368	CNN2	335.60	19.03	173.58	10.47	1.51E-05	calponin 2
204086_at	NM_006115	PRAME	7.54	1.80	40.93	6.11	1.44E-06	preferentially expressed antigen in melanoma
204271_s_at	M74921	EDNRB	385.40	106.32	2757.37	286.01	1.25E-09	endothelin receptor type B
205051_s_at	NM_000222	KIT	111.95	24.27	926.32	91.49	8.58E-09	v-kit Hardy-Zuckerman 4 feline sarcoma viral oncogene homolog
208073_x_at	NM_003316	TTC3	829.04	46.89	2365.96	180.22	3.50E-05	tetratricopeptide repeat domain 3
208073_x_at	NM_003316	TTC3	829.04	46.89	2365.96	180.22	3.50E-05	tetratricopeptide repeat domain 3
209168_at	AW148844	GPM6B	50.25	7.43	175.28	18.56	1.69E-07	glycoprotein M6B
212038_s_at	AL515918	VDAC1	963.19	52.79	1257.05	81.82	1.26E-05	voltage-dependent anion channel 1
216479_at	AL356414	RPL21	26.99	1.11	21.70	0.95	1.56E-04	ribosomal protein L21
217738_at	BF575514	NAMPT	41.75	8.88	175.46	18.24	7.90E-06	nicotinamide phosphoribosyltransferase
221951_at	AI739035	TMEM80	80.82	5.05	44.26	5.65	1.77E-05	transmembrane protein 80
222297_x_at	AV738806	RPL18	52.29	1.66	40.59	1.55	9.57E-06	Ribosomal protein L18
224991_at	AI819630	CMIP	66.89	7.30	26.92	4.93	1.69E-07	c-Maf-inducing protein
228156_at	AW342078	BC020163	53.52	4.05	33.38	6.11	5.60E-05	Homo sapiens, clone IMAGE:4346533, mRNA
244829_at	N44676	MGC40222	23.68	9.23	376.13	45.89	4.60E-11	Hypothetical protein MGC40222

Probeset ID: Probeset ID from Affymetrix U133 plus 2.0 array

GeneBank: GeneBank accession number

Nevi-mean: average expression value of nevi samples

Nevi-StdErr: standard error of nevi samples

Melanoma-mean: average expression value of melanoma samples

Melanoma-StdErr: standard error of melanoma samples

p-value: t-test ($p < 0.05$, Westfall and Young permutation multi-testing correction, false discovery rate, $q<0.05$)

Data S1. Details of Strategy for Melanoma Class Prediction Modeling

Array data containing 76 melanomas and 126 naevi were processed and normalized using GCRMA from Bioconductor (http://www.bioconductor.org). Gene targets with an expression value <100 across all 202 samples was filtered out from further consideration, leaving 22,526 genes for further analysis. The samples were then divided into a training dataset of 37 melanomas and 37 naevi and a test dataset of 39 melanomas and 89 naevi.

The training set data were then analyzed for differential gene expression by means of t-test with multi-testing correction (Westfall & Young Permutation method[1])($p < 0.05$, FDR < 0.05). Starting with the 22,526 genes, 422 differentially expressed genes were identified.

The 422 genes from the training dataset were further analyzed by stochastic gradient boosting method[2, 3] (TreeNet [Salford Systems, Inc.]) for class prediction modeling. The performance of each model was subsequently evaluated on the test dataset of 39 melanomas and 89 naevi. The following parameters were used for the class model building: learn rate of 0.001, subsample fraction of 0.5, influence trimming factor of 0.1, M-regression breakdown of 0.9, and cross entropy (likelihood) for the optimal logistic model selection criterion. The number of trees used for model building was set to 10,000, the maximum number of nodes per tree was set to 6, the minimum number of training observations in terminal nodes was set to 10, the maximum number of most-optimal models to save summary results for was set to 1 and a threshold of 0.5 was set for the classification modeling as the default setting for TreeNet.

With the TreeNet ranked variable importance cut-off value of >3.0, a class prediction model was generated from the training dataset that contained 168 of the 422 genes. This model correctly identified all 37 melanomas and 35 out of 37 naevi. The performance of this class prediction model was evaluated with the test data of 39 melanomas and 89 naevi. All 39 melanomas in the test dataset were accurately identified and 78 out of 89 naevi were called correctly by the 168-gene classifier, indicative of 100% sensitivity and 88% specificity.

We then sought to reduce the number of genes in this class prediction model while maintaining the model's high sensitivity and specificity. Starting with the 168 genes, the ranked variable importance cut-off value was set to >8.0. The resulting class prediction model contained 56 genes. Testing with the independent dataset revealed this 56-gene classifier to be 100% sensitive and 88% specific. This process was repeated using the 56-gene classifier in which the variable importance cut-off value was set to >10.0. The resulting 42-gene classifier, when tested, was again found to have a sensitivity of 100% and 88%, respectively.

To further reduce the number of predictors from the 42-gene model, we used a shaving method to generate by means of TreeNet progressively smaller models. Shaving removed the lowest ranked gene predictor from the existing TreeNet model such that the 100% sensitivity and 88% specificity was maintained upon testing. This shaving strategy eventuated in a predictive model containing the 17 genes that was found to have 100% sensitivity and 88% specificity upon testing.

1. Westfall PH and Young SS (1993) Resampling-Based Multiple Testing: Examples and Methods for p-Value Adjustment. Wiley, New York.
2. Friedman JH. Greedy Function Approximation: A Gradient Boosting Machine, Annals of Statistics. 2001; 29: 1189-1232.
3. Friedman JH. Stochastic Gradient Boosting. Computational Statistics & Data Analysis. 2002, 38: 367-378.

Schema for Melanoma Class Prediction Modeling

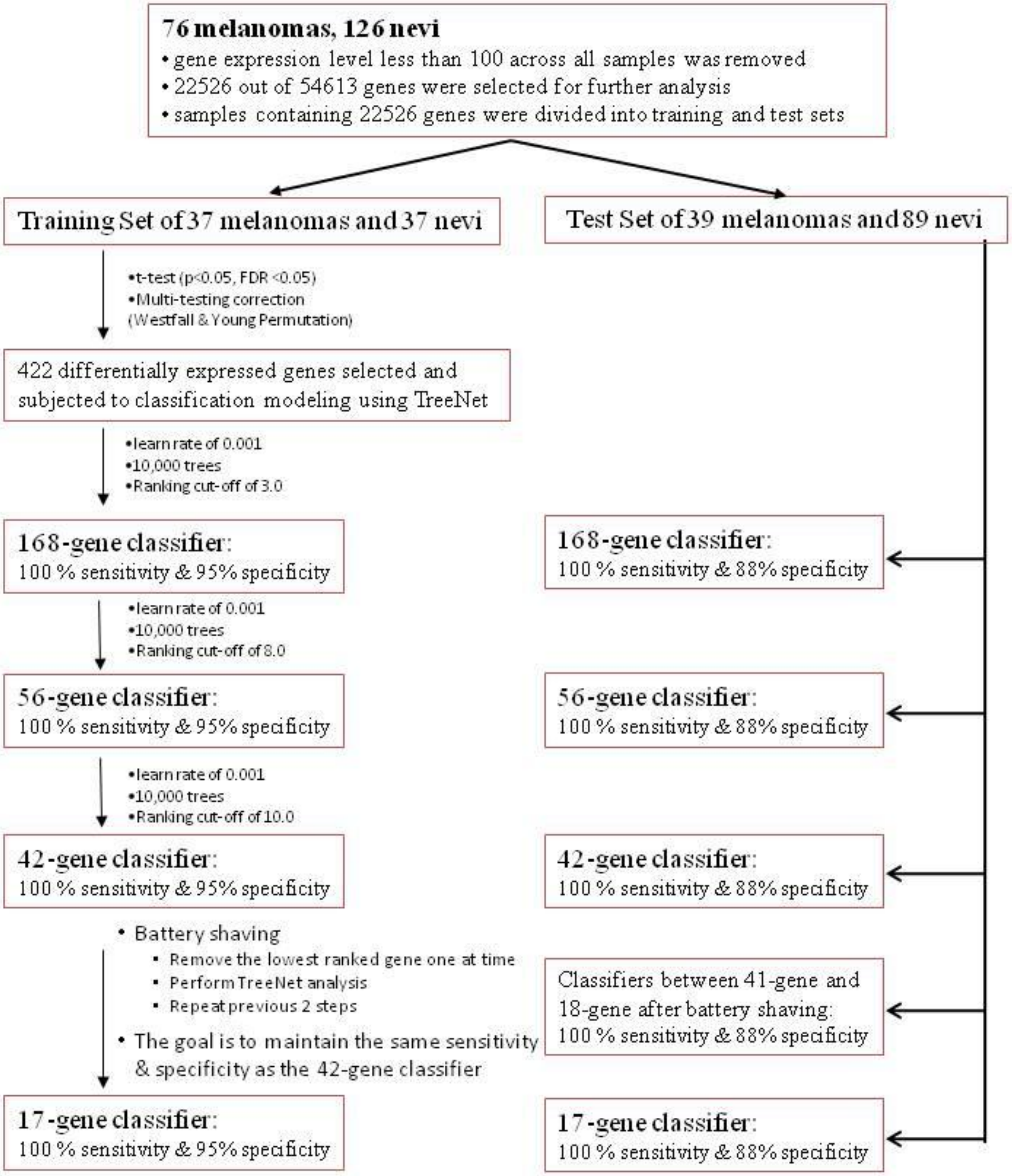

Data S2. Assay of melanoma and non-melanoma specimens by qRT-PCR using the 17-gene classifier recapitulates microarray results

The 17-gene classifier discovered by microarray analyses of EGIR tape-stripped specimens was evaluated by quantitative reverse transcription polymerase chain reaction assay (qRT-PCR).

Materials and Methods:

Predesigned TaqMan gene expression assays for each of the 17 genes and an internal control gene (mitochondrially encoded NADH dehydrogenase 6, MT-ND6), as well as TaqMan Universal PCR master mix, were purchased from Applied Biosystems (ABI), Foster City, CA.

Approximately 1.0 ng of EGIR-harvested RNA from 10 melanomas and 10 naevi was amplified using the Ovation FFPE RNA Amplification System (NuGEN Technologies, Inc., San Carlos, CA). Measurement of gene expression for each of the 17 genes in the classifier and an internal control gene (MT-ND6) were performed in triplicate by TaqMan-based qRT-PCR using 5 ng of amplified specimen on an ABI Prism 7900HT system.

Data processing and analysis:

The mean Ct value from triplicates of each of the 17 genes was normalized with the MT-ND6 internal control gene, based on the calculation of $2^{-(\text{CtgeneX} - \text{Ct MT-ND6})}$ for each sample.

Cluster analysis of resultant qRT-PCR data was performed according to Eisen et al.[1] Data were first log2 transformed and then median centered for genes and samples. These normalized data were further analyzed by the self organizing map algorithm[2] and then clustered with Spearman rank correlation similarity metrics.

Results:

Based on the 17-gene classifier, the preliminary qRT-PCR results (shown below) found that all 10 melanoma specimens were correctly called melanoma, while 9 of the 10 naevus specimens were called correctly – one being a false positive. These results in terms of sensitivity and specificity for melanoma detection are quite similar to those found with the test dataset (see Table 3 in the primary Results section of the paper) that were produced using GeneChip microarrays.

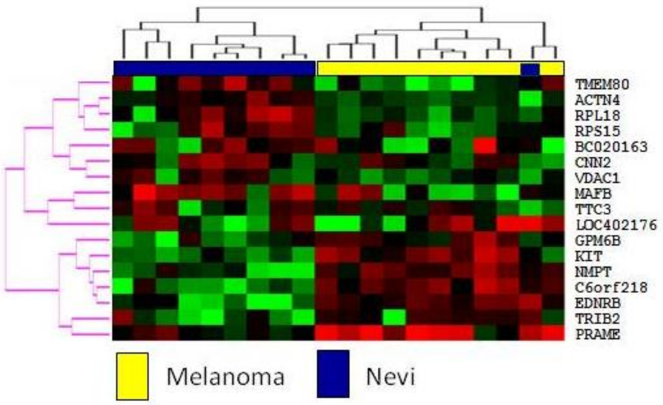

1. Eisen MB, Spellman PT, Brown PO, Botstein D. Cluster analysis and display of genome-wide expression patterns. *Proc Natl Acad Sci USA* 1998; **95**: 14863–14868.

2. Tamayo P, Slonim D, Mesirov J, et al. Interpreting patterns of gene expression with self-organizing maps: methods and application to hematopoietic differentiation. *Proc Natl Acad Sci USA* 1999; **96**: 2907–2912.
